# Balancing the trade-offs between land productivity, labor productivity and labor intensity

**DOI:** 10.1007/s13280-023-01887-4

**Published:** 2023-06-27

**Authors:** Cristina Chiarella, Patrick Meyfroidt, Dilini Abeygunawardane, Piero Conforti

**Affiliations:** 1 Earth and Life Institute, UCLouvain, Place de l’Université 1, 1348 Louvain-la-Neuve, Belgium; 2 Fonds de la Recherche Scientifique F.R.S.-FNRS, 1000 Brussels, Belgium; 3 Structural Development of Farms and Rural Areas (Structural Change), Leibniz Institute of Agricultural Development in Transition Economies (IAMO), Theodor-Lieser-Str. 2, 06120 Halle, Germany; 4 Statistics Division, Food and Agriculture Organization of the United Nations (FAO), Viale delle Terme di Caracalla, 00153 Rome, Italy

**Keywords:** Agricultural productivity, Farm size, Labor intensity, Policy decision making, Technical efficiency

## Abstract

Agricultural intensification, through increased yields, and raising incomes, through enhanced labor productivity, are two dimensions prioritized for sustainable agricultural development. Prioritizing these two outcomes leaves labor intensity as a hidden adjustment variable. Yet, when agriculture is mainstay and the prospects of labor absorption in other sectors are scarce, the density of agricultural employment is central for livelihoods. We revise relationships of land and labor productivity and labor intensity with farm size, using standardized data for 32 developing countries. We show that labor productivity increases with farm size, while land productivity and labor intensity decrease with farm size nonlinearly. Technical efficiency increases with farm size. We further systematize the evidence on how, beyond the farm level, local contexts can be pivotal in choosing how to prioritize the dimensions of the trade-off space. Our findings contribute to debates on the fate of small-scale farmers, and call for contextualized decisions.

**Supplementary Information:**

The online version contains supplementary material available at 10.1007/s13280-023-01887-4.

## Introduction

Land use is at the nexus of many key sustainability challenges including food security, poverty alleviation, nature conservation, and climate change mitigation. On the one hand, sustainable agricultural intensification (i.e., an increase in land productivity in ways that are environmentally and socially sustainable) is key to reducing human pressures on natural habitat, biodiversity, carbon stocks, and other ecosystem services. On the other hand, sustainable agricultural development should benefit local communities and provide them with adequate livelihoods (i.e., an increase in labor productivity). At the farm level, the goals of sustainable agricultural development, such as formulated in the Sustainable Development Goals, are hence to increase land productivity in order to spare land, and to increase labor productivity in order to increase economic returns for farmers. Yet, in regions where agriculture remains the main source of livelihood, particularly when prospects for absorption in the nonfarm sector are thin, spreading the benefits of farming across a larger number of households to sustain their livelihoods and lift them out of poverty is also a key goal of sustainable development. This goal can be captured effectively by indicators of labor intensity, or labor demand, i.e., the number of workers or working hours per hectare per year, which is a key indicator on how agriculture can contribute to create employment and livelihood opportunities for many, and especially for landless, unemployed, or underemployed farm workers. But, when the focus is on land and labor productivity, labor intensity is in fact a hidden dimension, implicitly considered as an adjustment variable, which results from goals set for land and labor productivity. In this study, we argue that balancing the trade-offs between the three dimensions of land productivity, labor productivity, and labor intensity is critical for sustainable land use and for achieving socially equitable forms of land sparing and agricultural development.

Among the multiple factors that influence this trade-off space, farm size is a central one. Farm size has historically been given a central place in agricultural growth. Seminal works have argued that initial factor proportion endowments (land to labor) and their price ratios explain further changes in factor proportions, which lead to alternative growth models, such as large farms coupled with mechanization in countries with abundant land and low population density, versus greater intensity of labor and bio-chemical technologies in countries with scarcer land (Hayami and Ruttan 1970). Similarly, Kislev and Peterson (1981) argued that increasing labor–capital price ratios lead to labor substitution and more mechanization adoption, which takes place in larger farms. Farm size is thus a key determinant of land and labor productivity as well as of labor intensity. Understanding the variability of labor intensity across different farm sizes or farming systems is thus pivotal in determining alternative agricultural development paths.

Changes in labor intensity have historically been addressed through intersectoral labor allocation. Rural transformation brought increases in labor productivity, displacing excess agricultural labor into the nonfarm sector. However, in some developing contexts, farm labor absorption by the nonfarm sector has been slow (Headey et al. 2010b). Such contexts call for striking a balance between increasing labor productivity and maintaining labor intensity in agriculture to avoid creating a large pool of unemployed or underemployed people. Paths of structural transformation have proceeded commonly with processes of farm consolidation and increases in farm size (which often occur in parallel with farm fragmentation and farm size reduction). Other factors such as crop types or forms of management also influence labor intensity, but are not dominant among global trends, as is the case of farm consolidation (Vittis et al. 2022). Hence, we focus on farm size because of its structural trend worldwide and its key role as a moderator of labor intensity and land and labor productivity.

To analyze the role of farm size, we evaluate how land productivity, labor productivity, and labor intensity vary with farm size, using a dataset of standardized agricultural indicators covering 32 countries. We evaluate these relationships both unconditionally and controlling for a variety of confounding factors. Such analysis presents stylized facts of the relationship between farm size and single-factor measures of productivity. To incorporate all factors of production jointly and complete the analysis of productivity trade-offs related to farm size, we also evaluate the effect of farm size on farms’ technical efficiency (TE), by estimating stochastic production frontier (SPF) regressions. Finally, building on these results on how land and labor productivity, labor intensity, and TE vary with farm size, we discuss how farm sizes relate to the prioritization of one of these dimensions, and the consequences of these relationships beyond the farm-size analysis. We also discuss several contextual factors that may favor the prioritization of one of the outcomes within the trade-off space over the other, and through which mechanisms.

## Background

### Labor intensity as the hidden dimension

Several long-run analyses weigh the effects of agricultural intensification, as a response to an increased food demand, and conservation outcomes, under different income and poverty projections (Adams et al. 2004; Wirsenius et al. 2010; Barrett et al. 2011; Tilman et al. 2011; Hasegawa et al. 2015; Grace et al. 2016; Liang et al. 2016; Popp et al. 2017; Springmann et al. 2018; Leclère et al. 2020; van Dijk et al. 2020; Williams et al. 2021) (SI, Table S1). In such studies, labor intensity and thus the number of agricultural workers is often neglected and de facto treated as an adjustment variable. The review in Table S1 frames the starting point of this article, as it shows the political relevance of leaving labor intensity to fluctuate freely to optimize outcomes of food production and conservation. A few other studies have recognized the absence of explicitly addressing labor supply in long-run analyses. In long-run crop, land use and price projections, agricultural inputs (labor, capital, fertilizer and other non-land inputs) are almost completely ignored in partial equilibrium (PE) models and mostly overlooked in general equilibrium (GE) models (Hertel et al. 2016). The omission of labor supply in such modeling hinders the assessment of changes in demand, winners and losers from changes in prices, and constraints on labor supply for agricultural production itself (Kuiper et al. 2020). The elasticity of supply of agricultural factors, including labor, is one of the most important contributors to future changes in prices, production, and land use (Hertel et al. 2016). With an increasingly skilled urban population, an increase in food prices would impact the urban poor consumers the hardest rather than benefit the rural poor agricultural producers (Hertel 2016; Kuiper et al. 2020).

Our contribution here is to include agricultural labor as an outcome that provides a source of livelihood, rather than only through its instrumental role in driving crop production, land use or price changes. Moreover, by comparing different farming systems, we argue that weighing the three outcomes of the trade-off space is context dependent, so addressing the role of labor intensity requires the combination of long-run projections, and microeconomic and case studies (as for example in Gibbon and Riisgaard 2014; Nolte and Ostermeier 2017; Baumert et al. 2019).

Ignoring labor intensity as a core objective together with land and labor productivity, especially within national programmatic strategies, may lead to treating individuals as “surplus population” (Li 2010). Li (2010) discusses this concept for the case of Asia, where large numbers of people in rural areas lost access to land encouraged by policies that promoted structural transformation, yet nonfarm jobs were insufficient to absorb the migrating population. Such a population is then considered a “surplus” that does not provide utility to capital. The places people inhabit and the resources there are utilized, but not them, hence people are detached from labor absorption.

**Fig. 1:**
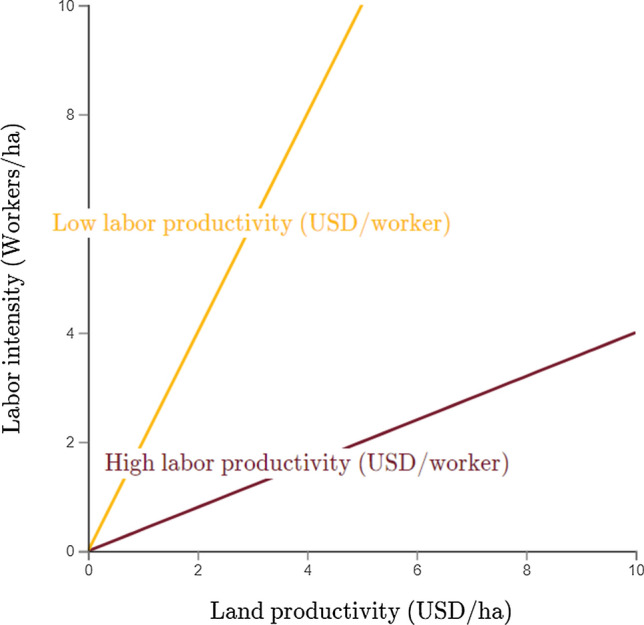
A graphical depiction of the three-dimensional trade-off space in two dimensions

Output per worker (or labor productivity) can be expressed as the identity:

1 $$\begin{aligned} {\text {Labor productivity}} = \frac{{\text {Land productivity}}}{{\text {Labor intensity}}} \end{aligned}$$

or:

2 $$\begin{aligned} \frac{Y}{L} = \frac{\frac{Y}{A}}{\frac{L}{A}}, \end{aligned}$$

where *Y* stands for agricultural output, *L* for the number of workers or amount of labor, and *A* for farm size. This identity can also be represented graphically (Fig. 1), with land productivity and labor intensity in the horizontal and vertical axis, respectively, and isolines for labor productivity. A central question of this study is where in this trade-off space do small, medium and large-scale farms stand.

### Farm size as a key determinant of land and labor productivity, labor intensity, and technical efficiency

Among the multiple factors that influence the position of different farming systems within the above trade-off space (Fig. 1), farm and field size play critical roles. Farm and field size are not identical but are often linked (Norton et al. 2009; Dannenberg and Kuemmerle 2010; Noack and Larsen 2019), and studies typically focus on either one or the other. An inverse relationship (IR) between farm size and land productivity has long been proposed and studied in the literature since the 1960s (Chayanov 1926/1986; Sen 1962; Carter 1984; Eswaran and Kotwal 1986; Barrett 1996). At the initial stage of this debate, it was generally conceived that larger farms were more productive than smaller farms, so that promoting small-scale farming would come at the expense of economic growth. When the evidence of the IR emerged, it favored both the promotion of small-scale farming for equity and efficiency gains, and arguments for land redistribution towards smaller farms for economic growth. Several empirical works have validated the IR, but others have also challenged it, finding no relationship (Kevane 1996; Zaibet and Dunn 1998), or attributing the IR to measurement error in self-reported data (Lamb 2003; Desiere and Jolliffe 2018), although some studies using GPS-based measures of land size have confirmed the IR (Carletto et al. 2013; Holden and Fisher 2013; Larson et al. 2014). Although these criticisms suggest that the productivity gains from small farms might not be real, they also show little or no evidence to disfavor small farms. The limited evidence of economies of scale is in the context of capital-intensive farming in high-income countries (Lund and Hill 1979; Kislev and Peterson 1991), but in a scale-neutral environment and under certain conditions (such as well-developed capital and insurance markets for example), large and small-scale farming could coexist with similar productivity levels, which is often overlooked in the polarization of this debate.

Several explanations have been provided for the IR, mainly related to market failures, since under perfect market conditions land productivity would be constant regardless of farm scale. One common explanation relates to labor market failures and labor supervision costs that lead small farmers to over-allocate family labor and other inputs on their own plots (Carter 1984; Eswaran and Kotwal 1986; Taslim 1990; Barrett 1996; Ali and Deininger 2015; Henderson 2015). Other explanations relate to differences in land quality, land and mechanization market failures (Wineman and Jayne 2021), or edge effects at the periphery of plots (Bevis and Barrett 2020).

Further explanations suggest that this relationship only holds across a certain range of farm sizes. A U-shaped relationship was postulated with larger commercial farms obtaining higher returns because of higher investment in capital and commercial crops (Carter and Wiebe 1990). The few studies that included a wide range of farm sizes have found a U-shaped relationship between field or farm size and agricultural productivity, considering yields and net value of output per ha, with the size thresholds where a positive relationship occurs at 5 ha (Jayne et al. 2019), 22 ha, or 11 ha for those medium-scale farmers that were previously small-scale farmers and expanded (Omotilewa et al. 2021). Further, the IR may be disappearing in several Asian countries where economic growth is inducing a switch from farm to nonfarm jobs and an increase in real wages; this is enabling farm expansion and the substitution of labor by machinery, which turns the effect of farm size on crop productivity positive for larger farms (Rada et al. 2015; Otsuka et al. 2016; Deininger et al. 2018; Liu et al. 2020).

This debate has been criticized for centering largely on a single-factor productivity metric, disregarding that high land productivity does not necessarily correspond to efficiency in inputs such as labor and capital (Morrison Paul et al. 2004; Headey et al. 2010a). Indeed, the relation between total factor productivity (TFP), defined as the ratio between aggregate output and aggregate inputs, and farm size, is context-dependent (Rada and Fuglie 2019). In Africa, greater productivity of smaller farms has been attributed to imperfections in labor and land markets, and TFP has been found to be greater for smaller farms (Julien et al. 2019). In Bangladesh, Gautam and Ahmed (2019) found a decreasing TFP with field size, although the strength of the relationship weakens over time. In Brazil, a relatively constant returns to scale relationship was observed in the mid-1980s, where larger farms reported slightly higher TFP levels; but by mid-2000s the relationship had become U-shaped (Rada and Fuglie 2019). These variations may relate to factor market transaction costs, economies of scale in farm mechanization, commodity specialization, or farm-size specific policies (Rada and Fuglie 2019; Foster and Rosenzweig 2022). In high-income countries, studies often find a higher TFP for larger farms (Key 2019; Sheng and Chancellor 2019). Besides farm size and managerial skills, several other factors may also influence single and TFP, as well as TE, including technological progress, geographical characteristics, type of crops, crop diversification, context or different policies (Adamopoulos and Restuccia 2014; Rada and Fuglie 2019).

## Materials and methods

### Data

This research relies on the Rural Livelihoods Information System (RuLIS) database (Food and Agriculture Organization 2022), a joint effort from the Food and Agriculture Organization (FAO), the World Bank and the International Fund for Agricultural Development (IFAD). We chose the RuLIS database because it is a collection of micro-data from National Representative Household Surveys, containing standardized and comparable indicators of agricultural variables, produced with similar methodology across countries. Large sample size and consistency in indicators across all sample countries make RuLIS data ideal for evaluating the relationships between land and labor productivity, labor intensity and farm size, as otherwise cross-country differences could be attributed to different methodologies in variables’ definition.

We extracted key variables on farm labor intensity, land and labor productivity, and farm size, as well as control variables to account for the inputs of the production function, households assets and other field management characteristics, for 32 countries for the land productivity estimates ($n=120\,394$), and 10 countries for the labor productivity and labor intensity estimates ($n=39\,356$). The countries included in the analysis are Albania, Armenia, Bangladesh, Bolivia, Bulgaria, Burkina Faso, Côte d’Ivoire, Ecuador, Ethiopia, Georgia, Ghana, Guatemala, India, Iraq, Kenya, Kyrgyzstan, Malawi, Mali, Mozambique, Nepal, Nicaragua, Niger, Nigeria, Pakistan, Panama, Peru, Rwanda, Serbia, Tanzania, Timor-Leste, Uganda, and Vietnam, with labor intensity data being only available for Burkina Faso, Ethiopia, India, Malawi, Mali, Niger, Nigeria, Panama, Tanzania, and Uganda. For each survey, we use the latest round available, with years ranging between 2005 and 2020. Table S2 (SI) summarizes the sources and years of the National Household Surveys.

The key variables for the analysis are: farm labor intensity (in number of working days per ha), land productivity (in 2017 USD PPP per ha), labor productivity (in 2017 USD PPP per working day), and farm size (in ha). Table S3 (SI) describes the main variables used in the analysis and their definitions. Farms are understood here as land that is arable or under permanent crops, owned or managed by the household. We conduct the analysis at the household level instead of the field level, as decisions about the farm, such as labor and input decisions, are primarily made at the household level. Our definition of farm scale is based solely on the physical size of land, we do not account for economic dimensions.^1^ All monetary variables are converted from local currency units (LCU) to 2017 USD PPP, using GDP per capita in constant LCU, GDP per capita in current LCU, and the GDP per capita PPP conversion factor, GDP (LCU per international USD); all from the World Development Indicators (WDI). The main outcomes (land and labor productivity, and labor intensity) had the 1% tails of their distributions trimmed. For the pooled sample of countries in the analysis, the median labor intensity across households is 247 working days per ha, median land productivity USD 1061 (2017 PPP) per ha, median labor productivity USD 3.4 (2017 PPP) per working day and the median farm size 0.8 ha. Most (52%) farmers in the sample are small-scale farmers farming plots between 0.2 and 1 ha. Farms larger than 10 ha represent 3% of the pooled sample.

### Empirical approach

We first evaluate the relationship between farm size and labor intensity, and land and labor productivity by estimating separate linear regressions, including a linear and a quadratic term for farm size. We control each of these estimations by demographic characteristics (age and education of household head, household size and household gender composition), area of residence, household services and assets,^2^ having livestock production, weather, price and disease shocks, and other non-agricultural income (social assistance, credit and off-farm income).

For consistency across estimations, all regressions use the same set of controls.^3^ The equations are estimated for the pool of countries (including country fixed effects^4^), and for each individual country (for their respective year). Equation 3 shows the specification for the land productivity estimation and for the pooled sample of countries.

3 $$\begin{aligned} \ln (y_{ic})=\beta _o +\beta _1 A_{ic}+\beta _2 A_{ic}^2+X_{ic}\gamma +\theta _c +\epsilon _{ic}, \end{aligned}$$

where $y_{ic}$ refers to the outcome variables (either land or labor productivity, or labor intensity), for household *i* and country *c*. *A* denotes farm size in ha, $\beta _1$ and $\beta _2$ are the coefficients of interest to evaluate the shape of the relationship. *X* is a vector of the control variables mentioned above, *c* are country fixed effects and $\epsilon$ household disturbances.

These linear regressions provide precise estimates, but looking at each of these relationships individually may disguise the overall productivity values when all factors of production are considered jointly. To account for all factors jointly, as a second step, we use a SPF model to estimate each household’s production function and simultaneously calculate the production frontier (the best-practice result of the production process) and TE (the productivity of each farm relative to the frontier) functions (Battese and Coelli 1995; Belotti et al. 2013). We assume a Cobb–Douglas production function. The stochastic production function takes the following form:

4 $$\begin{aligned} \ln (Y_{ic}/A_{ic})&=\beta _0 + \beta _1 \ln (A_{ic})+\beta _2\ln (L_{ic}/A_{ic})\nonumber \\&\quad +\beta _3 \ln (I_{ic}/A_{ic}) +\beta _4 K_{ic}+\theta _c\nonumber \\&\quad +[V_{ic}-U_{ic}(A_{ic},Z_{ic})], \end{aligned}$$

where $Y_{ic}/A_{ic}$ is land productivity or the value of output per ha for farm *i* and country *c*, and $A_{ic}$ is farm size in ha. $L_{ic}/A_{ic}$ encompass all labor inputs per ha, which include the number of adult family workers per ha (following Gautam and Ahmed 2019) and expenditure for hired labor per ha.^5^$I_{ic}/A_{ic}$ denote other inputs per ha, for which we consider seed costs (traditional and improved seed expenditure), and other input costs (such as inorganic fertilizers and other chemicals expenditure). $K_{ic}$ denotes fixed capital inputs, such as dummies for mechanization and irrigation, and livestock units. $\theta _c$ denotes country fixed effects which are included for the estimation for the pool of countries, and $V_{ic}$ represent random disturbances.

$U_{ic}$ is the inefficiency term of the SPF, which is estimated simultaneously in one step through Maximum Likelihood for unbiased and consistent estimates, assuming a truncated normal distribution, as typically assumed (Stevenson 1980). It measures TE for each farm, or the radial difference between the farm’s observed output compared to the potential output the farm could’ve obtained if producing at the best-practices frontier, given the inputs. This term ranges from 0 to 1, where 0 is total inefficiency and 1 is 100% efficiency. As possible factors that influence the inefficiency component and explain the variability of TE across households, we include farm size $A_{ic}$ in log form, and a vector of demographic characteristics $Z_{ic}$ to account for managerial capacity, which include age and education of the household head, and household composition. To explore regional dynamics, we also control for tertiles of land concentration at the regional level (calculated using the Herfindahl–Hirschman Index, HHI) and tertiles of nonfarm labor (defined as less than 30% of total income coming from agriculture) out of total employment at the regional level. The parameter of interest is the coefficient of farm size on the inefficiency function, as it directly tests the relationship between farm size and changes in TE.

Although the RuLIS data set provides a unique opportunity for harmonized global comparison, some limitations are worth highlighting. First, we are using cross-sectional data, which provides a snapshot comparison of different farm sizes within countries (and across countries for the pooled estimation), in contrast to assessing the effect of size changes in a given farm over time, if we were to use a panel data set. The drawback of using cross-sectional data is that it does not account for observed and unobserved factors that are not controlled for, such as contextual factors that can affect the relationship between the outcomes of interest and land size for different farms. Although following the same farms over time would more adequately account for such factors and improve the precision of the estimates, this goes beyond the available data and the goals of this study. Our goal is descriptive, to show the different characteristics between farm sizes, which have consequences at a broader level, for example, the density of employment provided at the regional level. Second, the year range of available national household surveys goes from 2005 to 2020. Even though this is a relatively narrow historical period, several contextual factors might still vary in this time range that may affect the relationships of interest in the pooled estimations. Third, as the surveys in the RuLIS data set focus on households, large farm enterprises are intrinsically under-represented, which limits the conclusions for the upper tail of the farm size distribution. Ideally, farm surveys would do a better job representing agriculture as a sector, but only household surveys are available for such a wide comparison across countries and with such socio-economic details. Yet, given that the majority of agricultural production is household-based, especially in low and middle-income countries, there is a large overlap between household surveys (whose data are collected in RuLIS) and agriculture as a sector.

## Results

**Fig. 2:**
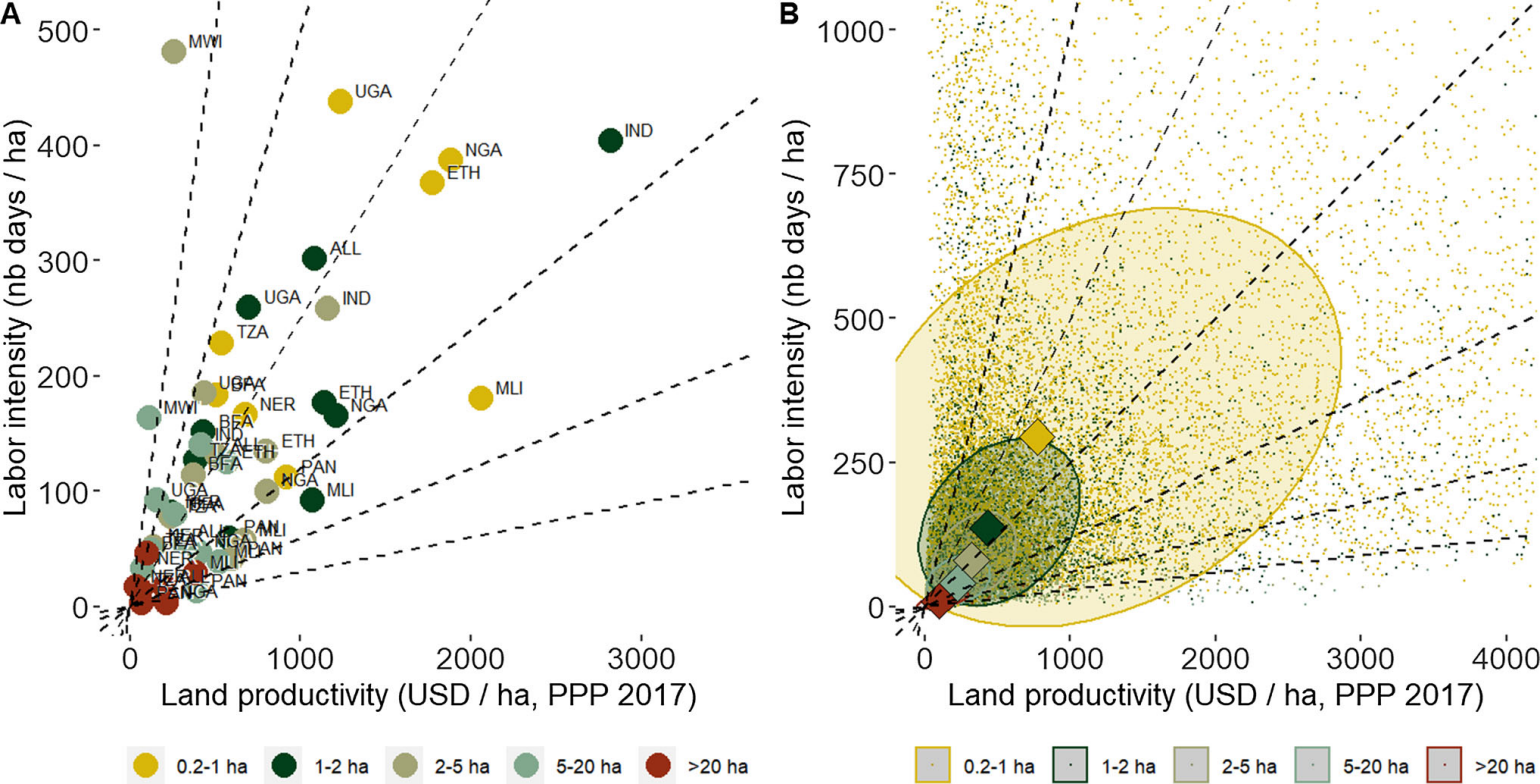
Labor intensity and land productivity by farm size category. *Notes* Figure 2**A** shows country median values of labor intensity and land productivity by farm size category (values in Table S4 in SI). Country codes are as follows: *ALL* All countries, *BFA* Burkina Faso, *ETH* Ethiopia, *IND* India, *MWI* Malawi, *MLI* Mali, *NER* Niger, *NGA* Nigeria, *PAN* Panama, *TZA* Tanzania, *UGA* Uganda. Figure 2**B** shows household levels of labor intensity and land productivity by farm size category. Squares show the sample median values by farm size category. The shape of the ellipses around each square represent a confidence region at the 95% level for the mean and covariance matrix of the distribution, indicating the covariance between land productivity and labor intensity

In line with the IR, smaller-scale farmers in our sample show greater levels of land productivity, as well as labor intensity (Fig. 2A and Table S4 in SI), supporting prior evidence (Carter 1984; Barrett 1996). As farm size increases, their median labor intensity and land productivity decrease. For farms between 0.2 and 1 ha, the median labor intensity is 886 working days per ha per year, and the median land productivity is USD 2736 (2017 PPP) per ha per year; whereas for farms greater than 20 ha, the median labor intensity is 18 working days per ha per year and the median land productivity USD 197 (2017 PPP) per ha per year. Although both decline with farm size, labor intensity declines faster than land productivity; and as farm size increases, median labor productivity shifts to isolines of higher labor productivity instead (Fig. 2B). Smaller farms also show a great variability of both labor intensity and land productivity (Fig. 2B), pointing to the important heterogeneity characterizing small farms, of which elucidating the causes require further investigation.

**Fig. 3:**
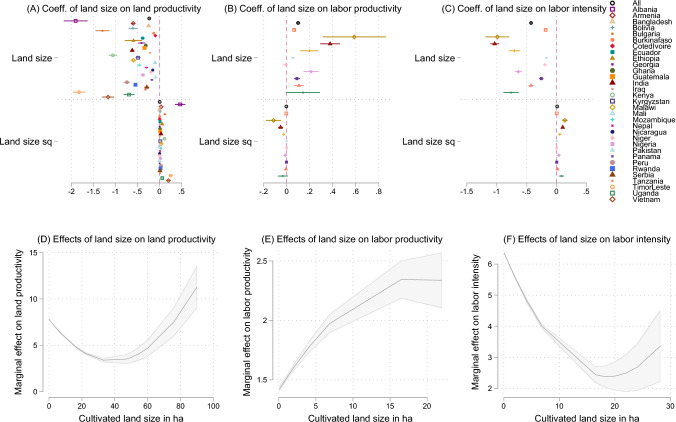
Coefficients from linear regressions on the linear and quadratic effect of land size on land productivity, labor productivity and labor intensity. *Notes* Land productivity, labor productivity and labor intensity are in natural logarithm. The 1% tails of the output variables are trimmed by country. Panels **D**, **E** and **F** show linear predictions of the outcome variable for marginal increases of cultivated land size, holding other variables constant at their mean

We then estimate the relationships between farm size and labor intensity, and land and labor productivity through the linear regressions described in the previous section. For both the pooled sample and individual countries, the IR between farm size and land productivity holds and is significant. Smaller farms show greater land productivity than larger farms (Fig. 3A and D), but the effect of farm size squared is positive, indicating a convex U-shaped relationship. The threshold at which the negative relationship becomes positive varies widely from 2 ha (for Albania), to 77 ha (for Côte d’Ivoire), with a median threshold across countries at 11 ha (consistent with Carter and Wiebe 1990; Jayne et al. 2019; Omotilewa et al. 2021). Omotilewa et al. (2021) find this turning point for Nigeria at 22 ha and an average turning point of 44 ha for the pooled sample. In contrast, as expected, larger farms show significantly greater labor productivity (Fig. 3B and E, with the exception of Niger and Uganda, where the relationship is not significant). The extent to which bigger farm size translates into greater labor productivity ranges from an increase of 2.7 percentage points (for Mali) to an increase of 18.7 percentage points (for Ethiopia) USD (2017 PPP) for each additional ha. Finally, for the pooled sample and all individual countries considered, larger farms significantly use less working days per ha for agricultural activities (Fig. 3C and F). A one-hectare increase translates into a decrease of working days that ranges between 10 percentage points (for Niger) to 57 percentage points (for Malawi).

Although the evidence for the non-linearity of these relationships is significant and consistent for most countries, robust characterization and interpretation of the upward curve of the U-shaped (or inverse U-shaped) relationship are lacking, as larger farms tend to be under-represented in household surveys (especially corporations involved in large-scale farming), which limits the statistical validity of the findings for such upper tail. The great dispersion on the inflection points of the relationships may also be partially explained by the smaller sample for larger farms, in addition to cross-country heterogeneities, crop types (with varying degrees of capital and labor intensity), and different policies and farming systems, among other factors that deserve further investigation with data that are more representative for larger farm sizes.

**Fig. 4:**
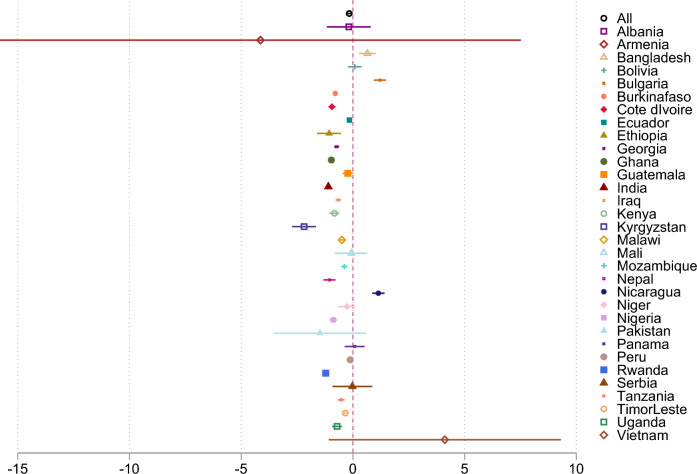
Coefficients of farm size on the inefficiency function of stochastic frontier estimations. *Notes* Figure shows the beta coefficients of the effect of farm size (in natural logarithm) to the inefficiency function of the SPF estimation on land productivity (in natural logarithm)

We then evaluate the degree of TE for each farm, by estimating SPF regressions. The results of the TE estimation show that, although the IR holds, TE increases with farm size in the pooled sample and in most individual countries (Fig. 4). Controlling for demographic characteristics that may affect farm management brings larger farms closer to the SPF than smaller farms. This holds true for most countries except for Albania, Armenia, Bangladesh, Bolivia, Bulgaria, Mali, Nicaragua, Niger, Pakistan, Panama, Serbia, and Vietnam, where the relation is insignificant or, in a few cases, reversed. Adding the indicators of land concentration and nonfarm labor to the inefficiency function (Table S10, SI) shows that high levels of land concentration do not only aggravate existing inequalities but are also detrimental for TE. Similarly, regions with a more vibrant nonfarm sector showed lower overall levels of TE, possibly as less people work in agriculture or consider agriculture their main occupation.

## Discussion

### From farm level to regional outcomes

We have focused the empirical analysis on understanding how the outcomes of the trade-off space vary with farm size because farm size is a critical dimension that is shifting globally along with structural transformation. Several other factors can also crucially influence land and labor outcomes, such as crop types, characteristics of the farm manager and of farm management, geography, and household wealth (Tables S5–S13, SI). These factors vary widely depending on the context and across specific farms, and can largely explain the wide heterogeneity in land and labor productivity and labor intensity seen within farm size categories. Yet, global trends of farm consolidation and farm fragmentation, which accompany structural transformation processes, and growing rural population densities such as in Sub-Saharan Africa (Mehrabi 2023), call for a specific attention to the role of farm size.

The empirical analysis has shown stylized facts for developing countries that confirm the strong relationship between farm size and key outcomes for sustainable development. As farm size increases, labor productivity and TE increase, but land productivity and labor intensity decrease, until a context-dependent threshold where larger farms start to see gains of land productivity. These relationships are typically observed from a farm-level perspective. Yet, beyond the farm level, the most favorable livelihood outcomes do not always stem from optimizing productivity. At the regional level, these results show that farm consolidation, through influencing agricultural yields, might influence land sparing outcomes, which could have critical consequences for expansion into natural areas and loss of biodiversity. This risk is especially problematic in the initial stages of the process of farm consolidation, since the relationship between farm size and land productivity turns positive after a threshold (which has a median value of 11 ha for all countries). Yet, the non-linear curve after this moderate size threshold suggests that a moderate level of farm consolidation beyond that threshold—still very far from a large-scale industrial farming model—might lead to land productivity gains (Foster and Rosenzweig 2022). Lower yields for larger farms do not imply that smallscale farming will result unequivocally in the most favorable outcomes. Farm fragmentation comes with efficiency costs, partly because of greater labor costs, which will reflect in higher food prices. Smaller farms also come at the expense of labor productivity and overall farm earnings. Even with greater yields, there’s a cap to how much a small farm can produce overall, which also raises the question of what size is too small for a minimum living earning.

Gains in labor productivity with greater farm sizes are part of the justification for structural transformation policies for poverty reduction. But labor intensity acquires particular relevance when moving beyond the farm-scale level, at the regional level, where the overall labor intensity of farming indicates the density of agricultural employment. With land consolidation, job opportunities whether for self-employed, wage farm workers, or family workers, decrease, which is especially problematic in contexts when there is surplus population not likely to be absorbed. The results also showed that labor intensity declines faster than land productivity, non-linearly, so the loss of labor demand at the regional level becomes even more problematic in the initial stages of land consolidation. Labor intensity might be a key priority for poverty reduction in areas with weak labor absorption, to avoid unemployment, underemployment and involuntary displacement.

### Context matters to balance trade-offs between land and labor productivity, and labor intensity

**Fig. 5:**
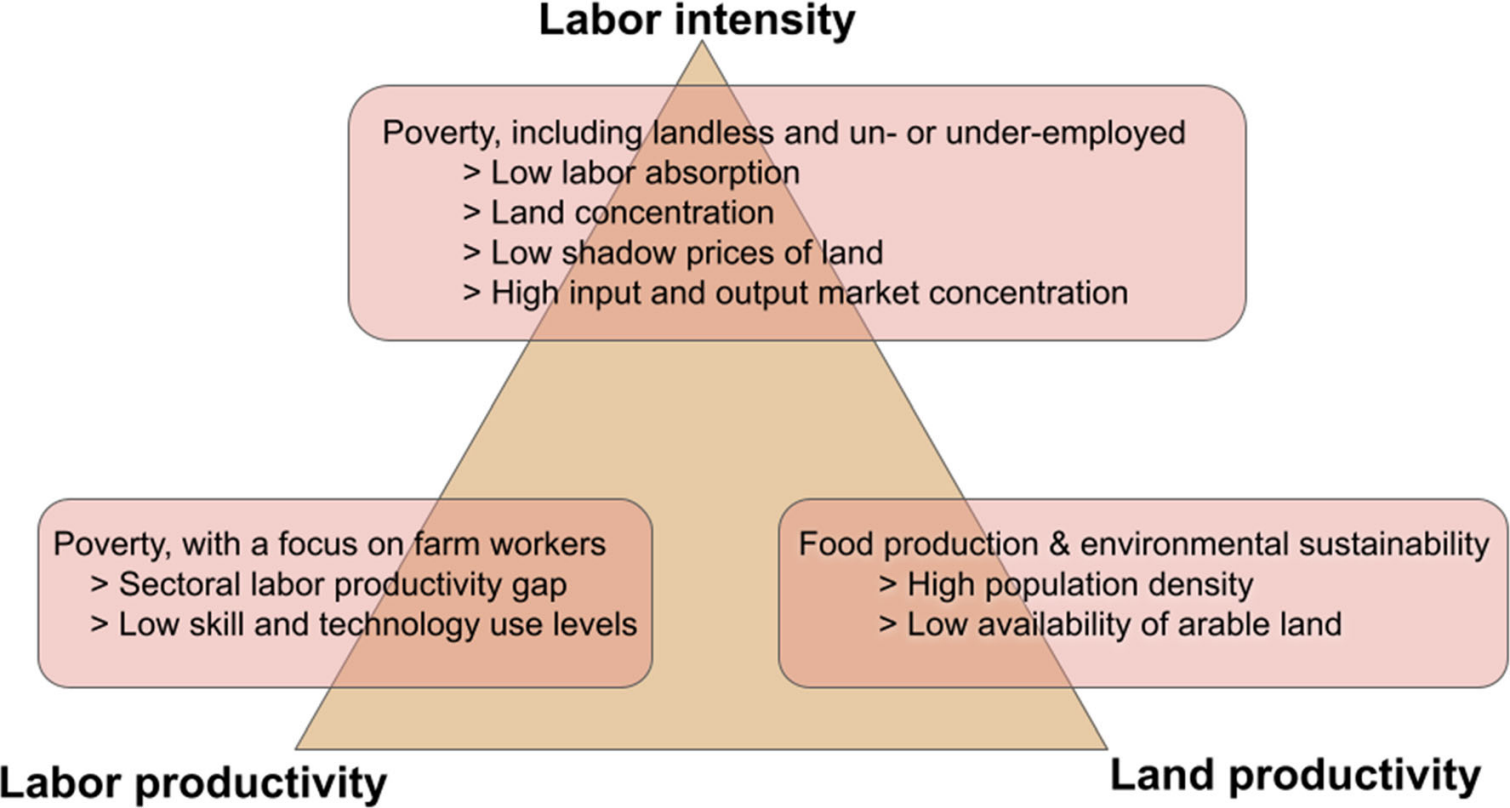
Contextual factors and proposed outcome of prioritization within trade-off space. *Notes* The three corners of the triangle are the three dimensions of the trade-off space introduced in Sect. 2. The title of the boxes linked to each dimension represent the objective to be prioritized. Items in the boxes represent contextual factors that may merit a prioritization of each dimension

Farm size has indeed a key role in sustainable development, but as this article demonstrates, advocating for specific farm sizes blindly from specific contexts is problematic because of the inherent trade-offs associated with farm size. In this section, we consider eight critical contextual factors and synthesize positive and normative arguments from the agricultural economics, rural development and sustainability literature, to inform when to prioritize one or another dimension within the trade-off space between land productivity, labor productivity and labor intensity. For each contextual factor, we discuss the advantages of prioritizing one of the dimensions of the trade-off space, and describe the mechanisms by which such factors can affect small, medium and large-scale farmers (Fig. 5).

#### Sectoral labor productivity gap

The historical process that has been accompanying economic development of nations has implied a reduction of farm labor and an increase in nonfarm labor, together with rural-to-urban migration. As described in Engel’s law, as the demand for non-food or non-agricultural goods increases at a faster pace compared to that of food and agriculture, the composition of production follows (leaving trade aside). Unless labor-saving innovations in the nonfarm sector are faster than in the farm sector, labor is expelled from agriculture. Technical change and policies directed to increase labor productivity in agriculture have also released labor from agriculture and promoted intersectoral reallocation of labor for significant economic growth (Timmer 2002, 2009; Barrett et al. 2010; Gollin 2010; Cao and Birchenall 2013). This structural transformation process has entailed large gaps in earnings or labor productivity between the nonfarm and farm sector (Young 2013; Gollin et al. 2014). These large inter-sectoral earning gaps have justified a historical focus on improving labor productivity in agriculture for poverty reduction.

Earning gaps have been largely attributed to imperfections in land and factors markets that impede the inter-sectoral reallocation of labor (Gollin 2021; Deininger et al. 2022). Consequently, policies have sought to address factor market imperfections, so that labor could freely move to regulate inter-sectoral labor misallocation and reduce general levels of poverty. Traditionally, however, these large inter-sectoral earning gaps have been estimated unconditionally and with cross-section data, and shown to decrease by a third when differences in human capital and hours worked were considered (Gollin et al. 2014). Further, using individual-level panel data, recent estimates have shown that when time-invariant characteristics of individuals are controlled for, productivity gaps for individuals that transition from farm to nonfarm labor are decreased by over 80% (Herrendorf and Schoellman 2018; Hamory et al. 2021). Much of the apparent labor productivity gaps are due to worker selection issues, meaning that more skilled laborers can self-select into nonfarm jobs that have higher labor productivity. This has important policy implications, as policies oriented to incentivize rural to urban migration may be less effective in decreasing poverty than traditionally thought, compared to raising labor productivity in agriculture.

#### Low skill and technology use levels

More skilled individuals are generally the most likely to end up in nonfarm jobs, and less skilled individuals are most likely to end up in farm jobs (Young 2013; Hamory et al. 2021). Furthermore, rural areas in low-income countries have the highest concentration of low skill levels and high illiteracy rates, especially in Sub-Saharan Africa (Beegle et al. 2016). This limits the possibilities for research and innovation in agriculture and poses a challenge for both the adoption of rapidly improving technologies and taking advantage of market opportunities. The lack of resources (land, water), poor physical and institutional infrastructure, and basic technology use (inputs and mechanization) also aggravate labor productivity stagnation (Barrett et al. 2017). Taken together, the evidence suggests that in contrast with a focus on facilitating rural to urban migration, policies can be more effective to address rural poverty by leveling-up human capital and technology use in rural areas, protecting farmers’ sources of labor and increasing labor productivity levels especially for smaller-scale agriculture.

#### Low labor absorption

The degrees of nonfarm labor absorption vary widely across the globe, even for regions with similar levels of economic development. Asia, for example, has experienced impressive rural poverty reduction attributed to nonfarm employment in rural areas and the strong linkages between the farm and nonfarm sectors; despite still experiencing slow job creation (De Janvry et al. 2005; Headey et al. 2010b). Africa, on the other hand, has seen great levels of agricultural exits, limited nonfarm job creation, and slow poverty reduction (Headey et al. 2010b). Nonfarm labor absorption also depends on the opportunities to develop mass manufactured industrial products. The pressures on the nonfarm sector to absorb agricultural surplus labor are even reinforced by the increasing population pressures and rapid rise of medium-scale farms in Africa (Jayne et al. 2014a). In such contexts of slow nonfarm labor absorption, policies oriented to prioritize labor intensity would be critical for poverty reduction or at least for mitigating poverty rise. Yet, the sheer number of aggregate agricultural labor is not the only relevant metric. Seasonality in agriculture has been found to be responsible of two thirds of rural underemployment (De Janvry et al. 2022), so an unrealized opportunity for poverty reduction lies in stronger linkages with the nonfarm sector that could provide local outside options to absorb the under-employed agricultural labor when out of the peak periods. Policies oriented to prioritize labor intensity could be most effective if tailored to local context and seasonality.

The development of capital-intensive industrial agriculture with low labor absorption together with gaps in public policy has also often generated little employment and few benefits for agricultural communities. The degree of labor absorption of capital-intensive agriculture depends on the level of mechanization, the types of crops, and the degree of vertical integration and inclusion of processing activities (Deininger and Xia 2016; Ali et al. 2017; Nolte and Ostermeier 2017; Mercandalli et al. 2021). The positive experiences of agro-export booms that have been documented occur when labor-intensive crops such as vegetables and fruits have been chosen, not only because they have high requirements of interactive labor (constant and careful choices over the planting and harvesting process), but also because they favor more contractual linkages that allow small-scale farmers to overcome constraints, offer two or three crops per season, or allow for crop mixes—a form of self-insurance (Carter et al. 1996; Van den Broeck et al. 2017; Fabry et al. 2022). Product and process differentiation also plays a key role in labor absorption. Typically organic farming and other highly specialized productions command more skilled labor, while promising higher returns, which is pulling many young workers back into agriculture in some contexts. Overall, in such settings of low labor absorption from large-scale industrial agriculture, prioritizing small-scale farming as a provider of labor intensity is key to support employment and livelihoods. In contrast, capital-intensive agriculture with low labor intensity could benefit local communities if population density is low, the likelihood of immigration is limited, and there is high labor absorption in the nonfarm sector (Deininger and Byerlee 2012).

#### Land concentration

The initial structure of land distribution shapes the long-run trajectory of agricultural change (Carter and Zimmerman 2000), with initial land concentration being self-reinforcing, possibly because of larger farms having greater power in land markets (Plogmann et al. 2020). High initial land concentration can lead to exclusionary growth, such as in the Latin American experience, where large private feudal estates, or more recently agro-export plantations, expanded production greatly but peasant farmers were often dispossessed of their land (Carter and Mesbah 1993; Jayne et al. 2014b). Bimodal land distribution systems tend to be detrimental for small farms, as buyers are able to obtain supplies from a small number of large-scale farms. To foster a distribution of income from agriculture across a larger number of people, market regulation policies might focus on labor intensity of small-scale farming as a source of agricultural growth. In fact, growth originating from agriculture has consistently been shown to have the greater impact on poverty reduction compared to growth from other sectors (Johnston and Kilby 1975; Lipton 2006; De Janvry and Sadoulet 2010; Hazell et al. 2010; Mellor 2014), which justifies a focus on labor intensity, not only to ensure that agriculture provides basic livelihoods, but also for overall growth and poverty reduction.

#### Low shadow prices of land

In many historical contexts, the provision of land to new investors at a price below its opportunity cost has encouraged land expansion rather than land intensification and often left communities with few or any benefits (Deininger and Byerlee 2012). In Africa, many of such new large-scale land acquisitions are owned by salaried urban new investors that live outside the area or by relatively privileged citizens in rural areas, less frequently by a process of accumulation of small-scale farmers (Sitko and Jayne 2014; Jayne et al. 2016). The ability to pay for land (the difference between prices and reservation prices of land) is a key determinant of agrarian transformation (Carter et al. 1996). Where small farms cannot offer upfront costs to buy land to participate in a booming agricultural sector, this can further reduce their access to land and the peasant sector. In a context with low shadow prices of land (or low competitiveness of small-scale farmers), and an abundance of new investors, a prioritization of labor intensity and of TFP can contribute to avoid further squeezing smallholders and maintaining small-scale agricultural activity as a source of livelihoods. In these contexts, policies to improve the productivity of small farmers may focus on facilitating the expansion of small-scale farms’ operations and their access to larger agricultural land and consolidation. Land market reform policies may be insufficient by themselves, as they could speed farm displacement. Such policies may have to be preceded by capital market reform (Carter and Zegarra 2000), that include capital and insurance provision, extension, and cooperative market arrangements. If labor productivity is to be targeted instead, through an export-oriented policy, access to capital and insurance for small-scale farmers must accompany such policies to ensure that they also benefit the rural poor (Barham et al. 1995; Carter et al. 1996).

#### High input and output market concentration

The increasing trends of market concentration of the seed and agrochemical transnational industries especially over the last two decades raise concerns for the majority of farmers as it can impact seed prices through weakened competition, limit choice (even more when there is vertical integration), and increase corporations’ bargaining power over working conditions and labor compensation (Clapp 2021). Farmers are also selling their products to increasingly concentrated output markets and intermediaries, often at low prices. Supermarkets require strict quantity and quality standards, flexibility for new requirements, consistent, traceable, and timely outputs, all which can disfavor small farms (Hazell et al. 2010). An increasing financialization of agro-food systems, where financial channels, shareholder values, and financial instruments are growing and occupying a bigger role (Meyfroidt et al. 2019), may also have consequences on prospects for small-scale producers, as they may be less likely to generate financial returns compared to corporate farmers. Supermarkets are also rising fast in Latin America (Reardon and Berdegué 2002) and parts of South-East Asia and China, although they are increasing at a slower rate in Africa and South Asia, where evidence shows that supermarket penetration increases with GDP per capita, income inequality, urbanization, female labor force participation and openness to foreign investment (Traill 2006). In such contexts of high input and output market concentration, institutional arrangements can compensate the increased costs and transaction costs for small farms (Hazell et al. 2010) and avoid displacing smaller-scale agriculture and its labor force further.

#### High population density

Evidence shows that high population pressure leads to outmigration, land fragmentation and land use intensification on small farms, which can contribute to land degradation (Holden and Otsuka 2014; Muyanga and Jayne 2014). With looming land scarcity, there is also major evidence that rising population density in Africa in particular is pushing for increased land intensification (Jayne et al. 2014a). Contexts with increasing population pressure call for policies focusing on sustainable increase in land productivity through interventions such as equitable access to land, infrastructure investment, soil conservation policies (e.g. payments for ecosystem services), land zoning, and market development.

#### Low availability of arable land

Arable land for further agricultural expansion is not as extensive as generally assumed, once constraints and trade-offs for land conversion are taken into account (Lambin et al. 2013; Meyfroidt et al. 2022). Furthermore, where such land is available, it is generally concentrated in a few countries or places, as in Africa for example (Deininger and Byerlee 2011; Chamberlin et al. 2014). Agricultural expansion into natural vegetation leads to biodiversity loss, carbon emissions, and warmer and drier conditions affecting local ecosystem services such as hydrological and nutrient cycles, carbon sequestration, soil fertility, and erosion control, among others (Shukla et al. 1990; Portela and Rademacher 2001; Lawrence and Vandecar 2015; Cohn et al. 2019; Costa et al. 2019). Expansion of large-scale farms are also likely to displace existing, small-scale farms. Beyond livelihood risks, the expansion of industrial agriculture into smallholder agricultural landscapes, or conventional intensification of smallholder systems, can also bring negative environmental impacts such as on-farm biodiversity loss (Ramankutty et al. 2019; Ricciardi et al. 2021), soil degradation, water contamination, heightened pest and disease attacks, and greenhouse gas emissions (Weis 2010; Woodhouse 2010). Increasing land productivity is a key element for reducing environmental impacts linked to natural habitat destruction, but developing environmentally-sustainable land uses requires to account for dimensions that go well beyond a simple focus on land productivity (Balmford et al. 2018; Kremen and Merenlender 2018; Meyfroidt et al. 2022).

## Conclusion

We started framing this article by showing how recent long-run analyses represent the trade-offs between land-saving outcomes (which support a land productivity prioritization) and on-farm economic outcomes (which support a labor productivity prioritization). In this approach, labor intensity is treated as an adjustment variable, in the sense that the overall labor intensity in the farm sector is determined based on outcomes on the other two dimensions. Based on (i) evidence on how these three outcomes vary across farm sizes and (ii) a synthesis on how these trade-offs play out beyond the farm level, we argue that labor intensity should be treated explicitly as an outcome of interest, since farm labor is a key source of livelihood and a safety net for the world’s poor, especially in contexts where agriculture is mainstay and nonfarm labor absorption is low.

The empirical analysis confirmed the well-established relationship, that smaller farms have greater land productivity than bigger farms up to a threshold that varies for each country, with a median value of 11 ha, after which the relationship between land productivity and farm size becomes positive, although cautious interpretation is needed for the upper tail of the farm size distribution due to low representativity of larger-scale farms. The thresholds at which a U-shaped relation appears between farm size and land productivity vary across countries between 2 and 77 ha. We also showed that smaller farms have greater labor intensity per ha, and that as farm size increases, labor intensity declines faster than land productivity. These relationships hold both unconditionally and conditionally. Smaller farms also showed greater dispersion in terms of labor and land productivity and labor intensity. The stochastic frontier estimations showed that TE increases with farm size, but also highlighted how some factors beyond the farm level are also important in explaining a higher technical efficiency. In particular, greater land concentration and a greater share of nonfarm labor at the regional level decrease technical efficiency. We discussed how achieving desired targets in terms of land and labor productivity, labor intensity, and efficiency might require promoting different farm sizes and models. Complementing these findings with a synthesis of the literature, we identified eight contextual factors beyond the farm level that matter for prioritizing the different outcomes in the trade-off space. Local contexts of low labor absorption (either from industrial agriculture or from the nonfarm sector), high land concentration, low shadow prices of land or low competitiveness of small farmers, low skill and technology use levels, and high input and output market concentration call for particular attention to labor intensity as a priority outcome in public policy. All these conditions are prevalent in many developing economies, particularly in Africa. The efforts to prioritize labor intensity in such contexts cannot deploy without suitable innovations in technologies and institutions, such that increases in labor intensity are matched with increases in labor productivity (as per the implications of Hayami and Ruttan 1970 for developing countries). Increases in labor productivity will also ensure that the increases in labor intensity can be supported through sufficient earnings.

Choosing the desired balance between the three focal dimensions is dependent on local contextual factors, and often implies trade-offs. Alternative farming systems such as agroecology often promise to avoid these tradeoffs by safeguarding farming activity through knowledge-intensive technologies (that also require skills improvement), with minimal damage to soil fertility, while increasing land and labor productivity (Fernandes et al. 2008; Kremen and Merenlender 2018; Akram-Lodhi 2021). Yet, empirical knowledge on labor productivity and labor intensity of agroecology and other forms of alternative agriculture remains scarce (Meyfroidt et al. 2019; Liebert et al. 2022).

The arguments presented in this article feed into current and historical discussions on the fate of small scale farming (Meyfroidt 2018). On the one hand, proponents of structural transformation, based on the cross-sectional urban–rural gaps in labor productivity, argue for policies that remove factor market barriers, so that workers can freely migrate to nonfarm jobs and level-up inter-sectoral labor misallocation. On the other hand, based on the IR and for normative reasons building on food justice and food sovereignty arguments, others advocate for protecting and enhancing labor-intensive small-scale farming. We showed that no single recipe is appropriate for all contexts and decision-makers should take local conditions into consideration to determine the right combination of policies. In certain contexts, in particular when there is no labor absorption or farmers have low levels of human capital, it is not suitable to push farmers out of agriculture, because alternatives are either absent or may even leave farmers worse off. In such situations, transforming farming activity in a way that does not push farmers out too rapidly, but enhances factor intensity, must be a part of the portfolio. This does not imply that labor-intensive farming is a long-term development path or in itself a solution to poverty.

This argument intends to demystify the debate based solely on farm size. Farm size indeed matters, but rather as a lever to navigate along the three dimensions of the trade-offs than for itself. Further, the dispersion of land and labor productivity and labor intensity for similar farm sizes is large, so that farm size cannot be the single policy target (Aragon et al. 2022). Along with farm size, many factors such as input and capital intensity, technologies, production systems, export-orientation and processing activities also matter in affecting the three outcomes. The dismissal of labor outcomes may also be pivotal in current debates on the digitalization of agriculture that overlook the demand for skilled labor potentially displacing unskilled labor. Attention to labor intensity as an outcome to prioritize may encourage the promotion of alternative activities, such as product and process differentiation, which can contribute to increased economic returns without displacing labor force, possibly even attracting new and young labor force.

Large-scale and long-run assessments of the future of food security and land use, whether through modeling including CGEs or IAMs, through statistical projections, or through qualitative evaluations and scenarios, would provide more relevant insights on sustainability by including labor intensity explicitly, not only as an input to improve model performance but as an outcome of interest. The available labor data needed to calibrate these models is often inadequate, but, as demand may also create supply, data may become more available when modelers make a convincing case for the need for it. Further, better understanding the future of food provision and sustainable land use requires not only aggregate long-run projections, but also a richer micro-level characterization, from local and regional case studies, of labor intensity across different farming models, and how these are influenced by policy interventions (Nolte and Ostermeier 2017; Baumert et al. 2019). For proper inference across the farm size spectrum, better representation of medium and large-scale farms, and samples that are followed across years, are also necessary in household surveys.

Our intention is to start a further discussion on how employment in agriculture and other land uses is often neglected in the current sustainability debates on land sparing, labor productivity, and land productivity; without even starting a broader discussion on the quality of labor (Weiler et al. 2016; Klassen et al. 2022). Livelihoods are fundamental to sustainability, and achieving the right balance between productivity, conservation goals and livelihoods is central to creating sustainable land use and achieving lasting sustainable development goals.

## Supplementary Information

Below is the link to the electronic supplementary material.

Supplementary file1 (PDF 238 KB)

## Data Availability

The data underlying this article are available in the FAO website at https://www.fao.org/in-action/rural-livelihoods-dataset-rulis/data-application/data/by-indicator/en. Codes are available at https://zenodo.org/record/7941182.

## Footnotes

1 The SDG monitoring framework for Target 2.3 has defined “small-scale” both in terms of its physical and economic size of the operations, and considers also livestock herds as a measure of scale (Methodology for computing and monitoring the sustainable development goal indicators 2.3.1 and 2.3.2 2019). For example, there are cases of pastoral communities with large land sizes that are still small-scale operations; or cases of quasi-landless operations with high capital intensity production.

2 That is, having electricity, running water, whether the household owns a home; included as a proxy for multidimensional poverty in rural areas.

3 Surveys were only considered as part of RuLIS if they allowed for the computation of most indicators, especially of those of income and productivity; but National household surveys use different questionnaires and methodologies, so not all surveys have the same questions available. There is an inherent trade-off between including more control variables per country versus including less control variables but estimate comparable specifications with the same control variables across a larger set of countries. Because we aim to contribute to comparable stylized facts, we opt for estimating the same specification across countries to the expense of including less control variables, although we also estimate more detailed specifications per country, which confirm that the results also hold when more precise estimates are obtained.

4 There’s no additional need to include year fixed effects, as these are collinear with country fixed effects since few surveys were made over the same year.

5 Most farmers in the sample are small-scale farmers and use little to no capital inputs and hired labor. Given that we are including these variables in their log form, to include farmers with no use of inputs in the estimations, we are adding a constant equal to one to the capital and labor inputs, such that these observations are included in the estimations. Such small constant values are only imputed to specific right hand side variables and not to outcome variables.
