# Supplementary material for: Balancing the trade-offs between land productivity, labor productivity and labor intensity
Source: Ambio. 2023 Jun 27;52(10):1618–34. doi: 10.1007/s13280-023-01887-4 (PMC10460764; doi:10.1007/s13280-023-01887-4)
Supplement: Supplementary file 1 — Supplementary file1 (PDF 238 KB) [file 13280_2023_1887_MOESM1_ESM.pdf]

Ambio

Supplementary Information

This supplementary information has not been peer reviewed

Title: **Balancing the trade-offs between land productivity, labor productivity and labor intensity**

Authors: Cristina Chiarella, Patrick Meyfroidt, Dilini Abeygunawardane, Piero Conforti

Background

Table S1 summarizes recent articles that use Integrated Assessment Models (IAMs) or other long-run analyses that weigh environmental and social outcomes.

Table S1: Review of recent literature that uses Integrated Assessment Models (IAMs) or similar to study the relationships between environmental and social outcomes

Reference	Title	Short summary
Leclère et al. (2020)	Bending the curve of terrestrial biodiversity needs an integrated strategy	Several simulation scenarios estimated through land use and biodiversity models to show how certain conservation policies and supply and demand effects could revert biodiversity loss trends by still enabling the provision of food for the human population.
Williams et al. (2021)	Proactive conservation to prevent habitat losses to agricultural expansion	Models of future agricultural land clearance based on historical land clearance, combined with species-specific habitat of 20k species. The paper concludes that policies should target how, where and what food is produced to prevent loss of species habitat and contribute to healthier human diets.
van Dijk et al. (2020)	Stakeholder-designed scenarios for global food security assessments	Analysis of how global food security could be affected in light of four simulated scenarios characterized by high and low levels of two dimensions: natural resources use and social equality.
Popp et al. (2017)	Land-use futures in the shared socio-economic pathways	Use of IAMs for projections of possible land-use changes and their consequences for the agricultural system, food provision, prices, and greenhouse gas emissions.
Springmann et al. (2018)	Options for keeping the food system within environmental limits	Through a global food systems model, this paper simulates how expected changes in population and income levels could increase the environmental effects of the food system. Options for reducing the environmental effects are also analyzed.
Hasegawa et al. (2015)	Scenarios for the risk of hunger in the twenty-first century using Shared Socioeconomic Pathways	Simulation of five scenarios of the Shared Socioeconomic Pathways (SSPs) using Integrated Model CGE and analysis of how each will affect future hunger risk. These scenarios differ in terms of "sustainability", "fragmentation", and combinations of these across high and low-income countries.
Wirsenius et al. (2010)	How much land is needed for global food production under scenarios of dietary changes and livestock productivity increases in 2030?	Scenarios of global land use for 2030 to investigate the potential of land-minimized growth of food supply. The scenarios are related to: efficiency in animal production, decreased food waste, and dietary changes.
Tilman et al. (2011)	Global food demand and the sustainable intensification of agriculture	Projections of global demand for crop production for 2050 and evaluation of the environmental impacts of different ways to meet this demand, in terms of intensification/extensification across richer and poorer nations.
Adams et al. (2004)	Biodiversity Conservation and the Eradication of Poverty	Review of the links between poverty alleviation and biodiversity conservation. Provides a conceptual typology with four links: i) poverty and conservation are separate policy realms, ii) poverty is a critical constraint on conservation, iii) conservation should not compromise poverty reduction, iv) poverty reduction depends on living resource conservation.
Barrett et al. (2011)	On biodiversity conservation and poverty traps	Preamble of special issue on empirical papers that explore link between biodiversity conservation and poverty traps. Papers in special issue are characterized as: i) those that explore the relationship between protected areas and poverty, and ii) new economic, social, and political approaches to achieve biodiversity conservation and social improvement.
Grace et al. (2016)	Integrative modelling reveals mechanisms linking productivity and plant species richness	Provides evidence, by analysing global grassland plots, that accumulation of biomass leads to a negative effect of species richness, species richness increases productivity, and climate and soils richness increase productivity.
Liang et al. (2016)	Positive biodiversity-productivity relationship predominant in global forests	Analysis in 44 countries that shows that a loss in biodiversity leads also to a loss in forest productivity.

Methodology

Data sources

Table S2 summarizes the sources of the National Household Surveys used for this analysis and compiled by the RuLIS dataset.

Table S2: National Household Surveys processed by RuLIS and used in the analysis

Country	Survey	Year	Source
Albania	Living Standard Measurement Survey	2005	Institute of Statistics of Albania (INSTAT)
Armenia	Integrated Living Conditions Survey	2013	National Statistical Service of the Republic of Armenia
Bangladesh	Household Income-Expenditure Survey	2010	Bangladesh Bureau of Statistics
Bolivia	Encuesta de los Hogares	2008	Instituto Nacional de Estadística - Ministerio de Planificación del Desarrollo - Bolivia
Bulgaria	Multitopic Household Survey 2007	2007	Gallup International
Burkina Faso	Enquête Multisectorielle Continue	2014/15	Institut National de la Statistique et de la Démographie - Ministère de l'Economie et des Finances
Cote d'Ivioire	Enquête sur le Niveau de Vie des Ménages	2008	Institut National De La Statistique (INS) - Ministere d'Etat, Ministere du Plan et du Developpement
Ecuador	Encuesta sobre Condiciones de Vida	2014	Instituto de Estadística y Censos
Ethiopia	Ethiopia Socioeconomic Survey	2019	Central Statistics Agency of Ethiopia (CSA) - Ministry of Finance and Economic Development
Georgia	Integrated Household Survey	2015	The State Department for Statistics of Georgia - GEOSTAT
Ghana	Ghana Living Standards Survey	2017	Ghana Statistical Service (GSS)
Guatemala	Encuesta Nacional de Condiciones de Vida	2014	Instituto Nacional de Estadística - Gobierno de Guatemala
India	India Human Development Survey	2012	National Council of Applied Economic Research, New Delhi
Iraq	The Iraq household socio-economic survey	2012	Organization for Statistics and Information Technology (COSIT) - Ministry of Planning, Government of Iraq
Kenya	Integrated Household Budget Survey	2005/06	Kenya National Bureau of Statistics
Kyrgyzstan	Integrated Sample Household Budget and Labor Survey	2013	National Statistical Committee of the Kyrgyz Republic - NSC
Malawi	Third Integrated Household Survey	2020	National Statistical Office (NSO) - Ministry of Economic Planning and Development (MoEPD)
Mali	Enquête Agricole de conjoncture integrée aux Conditions de Vie des Ménages	2017	Cellule de Planification et de Statistiques - Ministère du Développement Rural Institut National de la Statistique - Gouvernement du Mali - Direction Nationale de l'Agriculture
Mozambique	Inquérito sobre Orçamento Familiar	2009	Direcção de Censos e Inquéritos - Instituto Nacional de Estatística (INE) - Ministry of Planning and Development
Nepal	Nepal Living Standards Survey	2011	Instituto Nacional de Estadística y Geografía
Nicaragua	Encuesta Nacional de Hogares sobre Medición de Nivel de Vida	2014	National Bureau of Statistics
Niger	National Survey on Household Living Conditions and Agriculture	2014	Survey and Census Division - National Institute of Statistics
Nigeria	General Household Survey	2019	National Bureau of Statistics (NBS)
Pakistan	Pakistan Social and Living Standards Measurement Survey	2013/2014	Federal Bureau of Statistics - Government of Pakistan
Panama	Encuesta de Niveles de Vida	2008	Instituto Nacional de Estadística y Censo (INEC)
Peru	Encuesta Nacional de Hogares	2019	Instituto Nacional de Estadística e Informática - República del Perú
Rwanda	Integrated Household Living Conditions Survey	2014	National Institute of Statistics of Rwanda - Ministry of Finance and Economic Planning
Serbia	Living Standards Measurement Survey	2007	Statistical Office of the Republic of Serbia
Tanzania	National Panel Survey	2015	National Bureau of Statistics
Timor-Leste	Survey of Living Standards	2008	National Bureau of Statistics
Uganda	The Uganda National Panel Survey	2016	Uganda Bureau of Statistics (UBOS)
Vietnam	Household Living Standards Survey	2010	General Statistics Office (GSO) - Ministry of Planning and Investment

Table S3 shows the main variables used in the analysis and their definition.

Table S3: Variables used in the analysis

Variable	Definition	Unit
Labor intensity	Total days of labor worked (family, hired and exchanged) for cropping activities by unit of farm size (cultivated)	Working days/ha
Land productivity	Total value of production from crop activities by unit of farm size (cultivated)	2017 USD PPP/ha
Labor productivity	Total value of production from crop activities by total working days in crop activities	2017 USD PPP/working days
Farm size	Area of cultivated land owned or cultivated by the household in ha	ha
Labor costs	Total expenditure for labor input for crop production on the last 12 months by unit of farm size (arable or permanent)	2017 USD PPP/HA
Family labor	Proxied by the number of HH members between 15 and 60 years old	Number of HH members
Seed costs	Sum of total expenditure on seeds and on improved seeds	2017 USD PPP
Other input costs	Sum of total expenditure on chemicals and and on inorganic fertilizer	2017 USD PPP
Mechanized equipment owned	Dummy for households owning mechanized equipment for farm activities	Dummy variable
Irrigation	Dummy for household using irrigation on cultivated land	Dummy variable
Tropical livestock units (TLU)	Total livestock number expressed in tropical livestock units	Number of TLUs
Education of HH head	Years of education of the HH head	Number of years
Age of HH head	Age of the HH head in years	Number of years
HH composition	Household composition categories (1 – only female adults, 2 – only male adults, 3 – male and female adults)	Categorical variable included as dummy variables
Urban	Urban dummy (0 – rural, 1 – urban)	Dummy variable
Electricity	HH with access to electricity (0 – no electricity, 1 - electricity)	Dummy variable
Running water	HH with water piped into the dwelling	Dummy variable
Owns home	Dwelling owned by the household	Dummy variable
Toilet	HH has access to improved sanitation facilities	Dummy variable
Livestock production	HH engages in livestock production during prior 12 months	Dummy variable
Social assistance	At least one HH member received social assistance transfers (cash, in-kind, public transfers, fee waivers, subsidies) in the prior 12 months	Dummy variable
Shocks	HH experienced weather, market price-related or disease related shocks in the prior 12 months	Dummy variable
Credit	HH received any loan in the prior 12 months	Dummy variable
Off-farm income	Total HH income derived from off-farm activities (non-ag self-employment, wages, transfers and other income)	2017 USD PPP

Results

Descriptive statistics

Table S4 shows country median values of labor intensity (in number of days per ha) and land productivity (in USD in 2017 PPP per ha). This is the information behind Figure 2A in the manuscript.

Table S4: Labor intensity and land productivity by farm size category

	Farm size category									
	0.2-1 ha		1-2 ha		2-5 ha		5-20 ha		20 ha+	
Country	Labor intensity	Land productivity	Labor intensity	Land productivity	Labor intensity	Land productivity	Labor intensity	Land productivity	Labor intensity	Land productivity
All	885.7	2735.7	302.2	1082.8	131.6	507.4	56.9	308.1	17.5	196.9
Burkina Faso	183.7	503	151.8	429.7	114.7	369.5	80.6	260.6	47.1	99.3
Ethiopia	367.7	1769.7	177.1	1138.7	134.7	797.8	125.6	565.4		
India	1014.7	4854.3	404.1	2814.8	258.5	1158.1	140.6	414.7		
Malawi	944	650.4	635.3	425.5	480.9	256.9	163.9	107.4		
Mali	180.9	2053.8	92.4	1065.3	57.6	669.3	39.5	520	29.6	380
Niger	166.8	675.2	82.5	237.2	52.8	137	33.9	72.7	17.4	32.6
Nigeria	387.5	1877.5	165.7	1207.2	100.7	801.1	48.1	407.7	4	210.6
Panama	112.6	916	60.3	579.6	41.8	598.5	13.8	387	3.6	65.7
Tanzania	229.2	537.2	127.4	381.4	78.8	238.4	49.7	121.2	14.6	57.6
Uganda	437.8	1233.4	259.5	696.2	185.6	433.4	92.7	154.3		

Notes: Table shows the information used for figure 2A in the manuscript. Labor intensity in number of days per ha, and land productivity in USD per ha, PPP 2017.

Land productivity

Tables S5-S7 show the full results of the linear estimation of cultivated land size and cultivated land size squared on land productivity for the pulled sample of countries and for each of the countries of the analysis. The inverse-relationship holds for all countries, but for all countries there is also a U-shaped relationship, where the average turning point is 44 ha (and a median of 11ha), but ranges from 2 ha (for Albania) to 77 ha (for Côte d'Ivoire)[1]

[1]To obtain the turning points, we follow the simple first order differentiation of the quadratic equation: $\delta(\beta_1 A_{ic} + \beta_2 A_{ic}^2)/\delta A_{ic} = 0$, holding other characteristics constant.

Table S5: Effects of land size on land productivity, Part I

	All	Albania	Armenia	Bangladesh	Bolivia	Bulgaria	Burkinafaso	CotedIvoire	Ecuador	Ethiopia	Georgia
Cultivated land size	-0.23***	-1.90***	-0.60***	-0.25***	-0.60***	-1.30***	-0.13***	-0.10***	-0.38***	-0.59***	-0.41***
	(0.00)	(0.14)	(0.03)	(0.02)	(0.05)	(0.08)	(0.01)	(0.00)	(0.02)	(0.10)	(0.04)
Cultivated land size squared	0.00***	0.47***	0.03***	-0.00	0.01***	0.12***	0.00***	0.00***	0.01***	0.06***	0.00***
	(0.00)	(0.06)	(0.00)	(0.00)	(0.00)	(0.02)	(0.00)	(0.00)	(0.00)	(0.02)	(0.00)
HH size	0.02***	0.02	0.03***	-0.00	0.06*	0.15***	0.03***	0.04***	-0.01	0.02	0.01
	(0.00)	(0.01)	(0.01)	(0.00)	(0.04)	(0.04)	(0.00)	(0.00)	(0.01)	(0.02)	(0.03)
Education HHhead	0.01***	0.02***	-0.00	0.00	-0.00	-0.00	-0.00	0.00	-0.01	-0.01	-0.00
	(0.00)	(0.01)	(0.01)	(0.00)	(0.02)	(0.01)	(0.01)	(0.00)	(0.00)	(0.01)	(0.01)
Age HHhead	-0.00**	0.00	0.00	-0.00	0.00	-0.02***	-0.00***	0.00*	-0.00	-0.00	-0.00
	(0.00)	(0.00)	(0.00)	(0.00)	(0.01)	(0.00)	(0.00)	(0.00)	(0.00)	(0.00)	(0.00)
Only male adults	0.11**	-0.37	-0.05	-0.11	0.16	0.08	0.40***	0.13**	0.14**	0.86***	-0.02
	(0.04)	(0.32)	(0.14)	(0.11)	(0.34)	(0.26)	(0.09)	(0.07)	(0.07)	(0.20)	(0.21)
Male female adults	0.18***	-0.25	0.14	0.05	0.21	0.35**	0.39***	0.29***	0.08*	0.43***	0.25*
	(0.02)	(0.25)	(0.10)	(0.04)	(0.21)	(0.15)	(0.04)	(0.05)	(0.05)	(0.16)	(0.15)
Urban	-0.10***	0.02	0.20**	0.03	0.03	-0.36***	-0.10***	-0.26***	-0.12***	0.00	-0.16
	(0.02)	(0.11)	(0.09)	(0.03)	(0.39)	(0.11)	(0.03)	(0.05)	(0.04)	(.)	(0.17)
Electricity	0.08***	0.00	-0.30	0.14***	-0.21	0.00	0.09***	0.01	0.05	0.11	-0.60***
	(0.02)	(.)	(0.22)	(0.02)	(0.15)	(.)	(0.03)	(0.03)	(0.15)	(0.08)	(0.17)
Running water	0.09***	-0.14***	0.08	-0.11	-0.98**	-0.05	-0.25***	-0.02	-0.19***	-2.26***	0.22***
	(0.02)	(0.05)	(0.10)	(0.09)	(0.39)	(0.23)	(0.08)	(0.03)	(0.04)	(0.28)	(0.08)
Owns home	0.10***	-0.05	0.04	0.24***	0.03	0.56**	-0.11***	0.13***	0.03	-0.08	0.34
	(0.03)	(0.19)	(0.27)	(0.05)	(0.25)	(0.25)	(0.04)	(0.03)	(0.04)	(0.19)	(0.28)
Toilet	0.05***	0.03	0.27***	0.00	-0.13	-0.17*	0.01	0.03	0.07	-0.14*	0.20
	(0.01)	(0.05)	(0.04)	(0.02)	(0.15)	(0.10)	(0.02)	(0.10)	(0.05)	(0.08)	(0.14)
Livestock prod.	0.07***	0.09	-0.12***	-0.02	0.49	0.20*	0.06***	0.07**	-0.03	0.73***	-0.03
	(0.01)	(0.15)	(0.04)	(0.03)	(0.34)	(0.11)	(0.02)	(0.03)	(0.32)	(0.18)	(0.14)
Off-farm income	-0.00	-0.00	-0.00	-0.00	-0.00	-0.00	-0.00	0.00***	-0.00	0.00	-0.00
	(0.00)	(0.00)	(0.00)	(0.00)	(0.00)	(0.00)	(0.00)	(0.00)	(0.00)	(0.00)	(0.00)
Country fixed effects	✓										
R^2	0.62	0.35	0.51	0.18	0.45	0.46	0.17	0.24	0.35	0.19	0.10
N	120394	1295	1309	4010	387	796	7153	5647	7559	672	1709

Notes: Standard errors in parentheses
$^*p < 0.10$, $^{**}p < 0.05$, $^{***}p < 0.01$.

Table S6: Effects of land size on land productivity, Part II

	Ghana	Guatemala	India	Iraq	Kenya	Kyrgyzstan	Malawi	Mali	Mozambique	Nepal	Nicaragua
Cultivated land size	-0.31***	-0.34***	-0.62***	-0.22***	-1.06***	-0.49***	-0.59***	-0.10***	-0.46***	-0.30***	-0.16***
	(0.02)	(0.02)	(0.02)	(0.01)	(0.05)	(0.03)	(0.03)	(0.01)	(0.02)	(0.02)	(0.02)
Cultivated land size squared	0.01***	0.01***	0.03***	0.00***	0.11***	0.02***	0.04***	0.00***	0.03***	0.01***	0.00***
	(0.00)	(0.00)	(0.00)	(0.00)	(0.01)	(0.00)	(0.01)	(0.00)	(0.00)	(0.00)	(0.00)
HH size	0.04***	0.01	0.01***	0.01**	-0.00	0.04**	0.00	0.01*	0.01**	0.06***	0.04**
	(0.01)	(0.01)	(0.00)	(0.01)	(0.01)	(0.02)	(0.01)	(0.00)	(0.01)	(0.01)	(0.02)
Education HHhead	-0.01**	0.01**	0.01***	0.01	0.04***	0.00	0.03***	-0.00	0.02***	0.02***	0.01
	(0.01)	(0.00)	(0.00)	(0.01)	(0.00)	(0.01)	(0.00)	(0.01)	(0.01)	(0.00)	(0.01)
Age HHhead	0.00***	0.00	0.00	0.00	0.00***	-0.00	0.00***	-0.00	-0.00	0.00***	-0.01
	(0.00)	(0.00)	(0.00)	(0.00)	(0.00)	(0.00)	(0.00)	(0.00)	(0.00)	(0.00)	(0.00)
Only male adults	-0.14*	0.08	0.03	-1.55***	0.20**	-0.05	-0.01	0.03	0.02	-0.31**	0.17
	(0.08)	(0.13)	(0.13)	(0.41)	(0.08)	(0.29)	(0.06)	(0.29)	(0.07)	(0.15)	(0.43)
Male female adults	0.12**	0.04	0.14***	-0.49**	0.26***	0.02	0.14***	0.49***	0.15***	0.16***	0.47
	(0.06)	(0.10)	(0.05)	(0.23)	(0.05)	(0.12)	(0.03)	(0.18)	(0.04)	(0.05)	(0.33)
Urban	-0.35***	0.05	-0.02	-0.49***	-0.30***	-0.12	-0.03	-0.51***	-0.09***	-0.02	-0.02
	(0.05)	(0.04)	(0.04)	(0.10)	(0.08)	(0.13)	(0.05)	(0.14)	(0.03)	(0.05)	(0.14)
Electricity	0.00	0.15***	0.11***	0.35***	0.06	-0.05	0.18***	0.23**	0.19***	0.13***	-0.04
	(.)	(0.03)	(0.03)	(0.12)	(0.08)	(0.28)	(0.05)	(0.09)	(0.06)	(0.03)	(0.12)
Running water	-0.06	0.07**	0.13***	0.09	0.29***	-0.38***	-0.10*	0.01	-0.11	0.04	0.11
	(0.10)	(0.03)	(0.02)	(0.06)	(0.05)	(0.07)	(0.05)	(0.09)	(0.07)	(0.04)	(0.14)
Owns home	-0.04	0.05	-0.14	-0.04	0.03	0.68**	0.08***	-0.26	-0.00	0.33***	-0.08
	(0.05)	(0.06)	(0.11)	(0.10)	(0.06)	(0.27)	(0.03)	(0.17)	(0.06)	(0.13)	(0.11)
Toilet	0.04	0.02	0.10***	0.12*	0.03	-0.21	0.04*	-0.13**	0.02	0.17***	-0.10
	(0.04)	(0.03)	(0.02)	(0.06)	(0.03)	(0.21)	(0.02)	(0.05)	(0.05)	(0.04)	(0.18)
Livestock prod.	0.25***	0.07**	0.03	0.03	0.36***	0.56***	0.23***	-0.14*	0.13***	0.08	0.05
	(0.04)	(0.03)	(0.02)	(0.06)	(0.05)	(0.07)	(0.02)	(0.09)	(0.03)	(0.06)	(0.16)
Off-farm income	-0.00***	-0.00*	-0.00***	-0.00**	-0.00**	-0.00***	-0.00*	0.00	-0.00	-0.00	0.00
	(0.00)	(0.00)	(0.00)	(0.00)	(0.00)	(0.00)	(0.00)	(0.00)	(0.00)	(0.00)	(0.00)
R^2	0.21	0.20	0.23	0.25	0.20	0.29	0.13	0.18	0.14	0.15	0.35
N	3773	3515	13303	2300	6494	1749	8280	2072	6785	3688	880

Notes: Standard errors in parentheses
$*p < 0.10$, $**p < 0.05$, $***p < 0.01$.

Table S7: Effects of land size on land productivity, Part III

	Niger	Nigeria	Pakistan	Panama	Peru	Rwanda	Serbia	Tanzania	TimorLeste	Uganda	Vietnam
Cultivated land size	-0.20***	-0.38***	-0.09***	-0.17***	-0.74***	-0.55***	-0.29***	-0.32***	-1.83***	-0.69***	-1.17***
	(0.01)	(0.03)	(0.01)	(0.02)	(0.04)	(0.02)	(0.03)	(0.02)	(0.07)	(0.06)	(0.07)
Cultivated land size squared	0.00***	0.02***	0.00	0.00***	0.03***	0.02***	0.01***	0.01***	0.25***	0.06***	0.20***
	(0.00)	(0.00)	(0.00)	(0.00)	(0.01)	(0.00)	(0.00)	(0.00)	(0.02)	(0.01)	(0.03)
HH size	0.01	0.04***	0.02***	-0.03	0.03***	-0.01	0.01	0.03***	0.04***	0.02**	0.05***
	(0.01)	(0.01)	(0.00)	(0.03)	(0.01)	(0.01)	(0.02)	(0.01)	(0.01)	(0.01)	(0.01)
Education HHhead	-0.03*	0.00	0.00	0.04	0.01**	0.02***	-0.02	0.02***	-0.02***	0.01	0.02***
	(0.01)	(0.01)	(0.00)	(0.03)	(0.00)	(0.00)	(0.01)	(0.01)	(0.01)	(0.01)	(0.00)
Age HHhead	0.00	-0.00*	-0.00***	-0.00	-0.00***	0.00**	-0.01*	-0.00	-0.01***	0.00	-0.00
	(0.00)	(0.00)	(0.00)	(0.01)	(0.00)	(0.00)	(0.00)	(0.00)	(0.00)	(0.00)	(0.00)
Only male adults	0.09	0.48**	0.36**	0.42	0.38***	0.20**	0.45**	0.21*	-0.25	0.18	-0.08
	(0.17)	(0.19)	(0.15)	(0.39)	(0.07)	(0.08)	(0.22)	(0.12)	(0.16)	(0.12)	(0.17)
Male female adults	0.14	0.39***	0.23***	0.24	0.42***	0.13***	0.47**	0.15**	0.04	0.21***	0.14**
	(0.10)	(0.14)	(0.07)	(0.36)	(0.05)	(0.04)	(0.19)	(0.07)	(0.11)	(0.07)	(0.06)
Urban	-0.10	-0.32***	0.32***	-0.99***	-0.12***	-0.13**	0.07	0.01	-0.12**	-0.19**	-0.14***
	(0.13)	(0.09)	(0.04)	(0.32)	(0.03)	(0.06)	(0.11)	(0.09)	(0.06)	(0.08)	(0.05)
Electricity	0.19	0.11*	0.09***	0.75***	0.16***	0.06	0.00	0.05	-0.17***	0.10	0.24***
	(0.13)	(0.06)	(0.03)	(0.15)	(0.04)	(0.04)	(.)	(0.11)	(0.05)	(0.12)	(0.06)
Running water	-0.46*	-0.30	0.24***	-0.07	0.05	-0.10	0.04	-0.10	0.07*	-0.09	-0.00
	(0.25)	(0.23)	(0.03)	(0.18)	(0.03)	(0.08)	(0.19)	(0.06)	(0.04)	(0.14)	(0.06)
Owns home	0.15	0.09	0.01	0.25	0.12***	0.22***	0.01	0.08	0.05	0.01	-0.18
	(0.13)	(0.07)	(0.04)	(0.29)	(0.05)	(0.05)	(0.19)	(0.08)	(0.06)	(0.12)	(0.18)
Toilet	0.38***	-0.13**	-0.03	0.20	0.04	0.13***	0.02	-0.05	0.05	0.28***	0.16***
	(0.08)	(0.06)	(0.02)	(0.15)	(0.03)	(0.03)	(0.13)	(0.06)	(0.06)	(0.07)	(0.03)
Livestock prod.	0.02	0.22***	0.12***	-0.65***	0.18***	0.36***	-0.26**	0.28***	0.02	-0.04	0.09***
	(0.07)	(0.06)	(0.02)	(0.21)	(0.05)	(0.03)	(0.10)	(0.06)	(0.04)	(0.05)	(0.03)
Off-farm income	0.00	0.00	-0.00***	-0.00*	-0.00***	0.00	0.00***	0.00	0.00	0.00	-0.00**
	(0.00)	(0.00)	(0.00)	(0.00)	(0.00)	(0.00)	(0.00)	(0.00)	(0.00)	(0.00)	(0.00)
R^2	0.32	0.21	0.20	0.30	0.38	0.13	0.31	0.24	0.52	0.14	0.23
N	1649	2346	4399	970	8296	7710	666	1759	2217	2204	4802

Notes: Standard errors in parentheses
$*p < 0.10$, $**p < 0.05$, $***p < 0.01$.

Labor productivity

Table S8 shows the effects of the same set of variables on labor productivity. The lack of agricultural labor information for all countries previously considered restricted the sample to only ten countries. Eight of the ten countries considered do show positive and significant effects of land size on labor productivity.

Table S8: Effects of land size on labor productivity

	All	Burkinafaso	Ethiopia	India	Malawi	Mali	Niger	Nigeria	Panama	Tanzania	Uganda
Cultivated land size	0.10***	0.07***	0.59***	0.38***	0.20***	0.05***	-0.01	0.21***	0.09***	0.11***	0.14*
	(0.01)	(0.01)	(0.14)	(0.04)	(0.04)	(0.01)	(0.01)	(0.03)	(0.01)	(0.02)	(0.08)
Cultivated land size squared	-0.00***	-0.00***	-0.11***	-0.05***	-0.03***	-0.00***	0.00	-0.01***	-0.00***	-0.01***	-0.03
	(0.00)	(0.00)	(0.03)	(0.01)	(0.01)	(0.00)	(0.00)	(0.00)	(0.00)	(0.00)	(0.02)
HH size	-0.03***	-0.01**	-0.02	-0.04***	-0.22***	-0.01***	-0.04***	-0.00	-0.08***	-0.05***	-0.04***
	(0.00)	(0.00)	(0.03)	(0.01)	(0.01)	(0.00)	(0.01)	(0.01)	(0.02)	(0.01)	(0.01)
Education HHhead	0.01***	0.02**	-0.01	0.02***	0.02***	-0.00	-0.01	-0.00	0.02	0.01	0.01
	(0.00)	(0.01)	(0.02)	(0.00)	(0.00)	(0.01)	(0.02)	(0.01)	(0.02)	(0.01)	(0.01)
Age HHhead	-0.00***	-0.00***	-0.01	-0.00	-0.01***	0.00	-0.00	-0.01***	-0.00	-0.01***	0.00
	(0.00)	(0.00)	(0.00)	(0.00)	(0.00)	(0.00)	(0.00)	(0.00)	(0.00)	(0.00)	(0.00)
Only male adults	0.27***	0.29***	1.43***	0.05	0.21***	0.12	-0.05	0.67***	0.22	0.29**	0.10
	(0.08)	(0.11)	(0.23)	(0.17)	(0.08)	(0.34)	(0.36)	(0.19)	(0.30)	(0.12)	(0.12)
Male female adults	0.08**	0.22***	0.45**	-0.00	0.01	0.28	0.08	0.38***	0.07	0.06	0.06
	(0.04)	(0.05)	(0.21)	(0.07)	(0.04)	(0.22)	(0.11)	(0.14)	(0.24)	(0.08)	(0.07)
Urban	0.02	-0.07*	0.00	0.08*	0.31***	-0.11	0.04	-0.13	-0.65*	0.05	-0.11
	(0.04)	(0.04)	(.)	(0.05)	(0.08)	(0.13)	(0.14)	(0.09)	(0.34)	(0.09)	(0.08)
Electricity	0.08***	0.18***	0.28***	0.03	0.19**	0.24***	0.10	0.08	0.88***	0.33***	0.19*
	(0.03)	(0.04)	(0.09)	(0.04)	(0.08)	(0.09)	(0.13)	(0.06)	(0.12)	(0.12)	(0.11)
Running water	0.18***	-0.08	-2.06***	0.14***	-0.10	0.04	0.19	0.21	-0.35**	0.04	0.29*
	(0.03)	(0.11)	(0.26)	(0.03)	(0.10)	(0.14)	(0.24)	(0.19)	(0.16)	(0.07)	(0.15)
Owns home	0.10**	-0.09*	-0.11	-0.07	0.05	-0.36**	0.01	0.07	0.60**	-0.05	0.10
	(0.04)	(0.05)	(0.26)	(0.12)	(0.05)	(0.18)	(0.17)	(0.07)	(0.25)	(0.08)	(0.12)
Toilet	0.10***	-0.07***	-0.20**	0.17***	-0.02	-0.12**	0.34***	-0.09	0.40***	-0.23***	0.23***
	(0.02)	(0.03)	(0.10)	(0.03)	(0.03)	(0.05)	(0.10)	(0.06)	(0.14)	(0.07)	(0.06)
Livestock prod.	-0.04*	0.05**	0.43**	-0.14***	0.04	-0.06	-0.16*	0.19***	0.27	0.18***	-0.10*
	(0.02)	(0.02)	(0.19)	(0.03)	(0.03)	(0.08)	(0.09)	(0.06)	(0.21)	(0.06)	(0.05)
Off-farm income	-0.00	0.00	0.00*	-0.00	-0.00	0.00	-0.00	0.00	0.00	0.00	0.00
	(0.00)	(0.00)	(0.00)	(0.00)	(0.00)	(0.00)	(0.00)	(0.00)	(0.00)	(0.00)	(0.00)
Country fixed effects	✓										
R^2	0.16	0.04	0.15	0.08	0.14	0.05	0.05	0.11	0.22	0.08	0.04
N	39356	7047	662	13094	8046	2045	1611	2063	882	1732	2174

Notes: Standard errors in parentheses
$^{*}p < 0.10$, $^{**}p < 0.05$, $^{***}p < 0.01$.

Labor intensity

Table S9 shows the last of the outcomes of the trade-off space regressed against farm size, again for the limited sample of countries with farm labor information. For all countries considered there is a negative and strong relationship between farm size and labor intensity.

Table S9: Effects of land size on labor intensity

	All	Burkinafaso	Ethiopia	India	Malawi	Mali	Niger	Nigeria	Panama	Tanzania	Uganda
Cultivated land size	-0.43***	-0.18***	-0.99***	-1.03***	-0.70***	-0.17***	-0.19***	-0.64***	-0.25***	-0.43***	-0.76***
	(0.01)	(0.01)	(0.10)	(0.04)	(0.05)	(0.01)	(0.01)	(0.03)	(0.02)	(0.02)	(0.06)
Cultivated land size squared	0.01***	0.00***	0.14***	0.10***	0.06***	0.00***	0.00***	0.04***	0.00***	0.02***	0.08***
	(0.00)	(0.00)	(0.02)	(0.01)	(0.01)	(0.00)	(0.00)	(0.00)	(0.00)	(0.00)	(0.02)
HH size	0.05***	0.03***	0.04**	0.05***	0.22***	0.02***	0.05***	0.04***	0.00	0.08***	0.06***
	(0.00)	(0.00)	(0.02)	(0.00)	(0.01)	(0.00)	(0.01)	(0.01)	(0.03)	(0.01)	(0.01)
Education HHhead	-0.00**	-0.02***	0.01	-0.00	0.01**	-0.00	-0.02*	0.01	0.01	0.02**	-0.00
	(0.00)	(0.01)	(0.01)	(0.00)	(0.00)	(0.01)	(0.01)	(0.00)	(0.02)	(0.01)	(0.00)
Age HHhead	0.00***	0.00**	0.01**	0.00**	0.01***	-0.00	0.00*	0.01***	0.00	0.01***	0.00
	(0.00)	(0.00)	(0.00)	(0.00)	(0.00)	(0.00)	(0.00)	(0.00)	(0.01)	(0.00)	(0.00)
Only male adults	-0.13***	0.06	-0.88***	-0.07	-0.22***	-0.09	0.09	-0.29**	0.32	-0.11	0.04
	(0.05)	(0.09)	(0.28)	(0.10)	(0.07)	(0.37)	(0.35)	(0.12)	(0.36)	(0.10)	(0.07)
Male female adults	0.10***	0.08**	-0.14	0.13**	0.11***	0.22	0.04	-0.06	0.29	0.10*	0.12**
	(0.03)	(0.04)	(0.18)	(0.06)	(0.04)	(0.25)	(0.09)	(0.08)	(0.34)	(0.06)	(0.05)
Urban	-0.14***	-0.08**	0.00	-0.13***	-0.34***	-0.53***	-0.18	-0.18***	0.12	-0.06	-0.07
	(0.03)	(0.04)	(.)	(0.04)	(0.08)	(0.11)	(0.11)	(0.06)	(0.34)	(0.07)	(0.05)
Electricity	-0.00	-0.08**	-0.16**	0.07***	-0.11	0.03	0.07	0.02	-0.14	-0.22**	-0.16*
	(0.02)	(0.04)	(0.07)	(0.02)	(0.08)	(0.08)	(0.12)	(0.04)	(0.16)	(0.09)	(0.10)
Running water	-0.10***	-0.16	-0.17	-0.05**	-0.05	-0.02	-0.48***	-0.23*	0.11	-0.16***	-0.49***
	(0.02)	(0.10)	(0.11)	(0.02)	(0.09)	(0.09)	(0.18)	(0.12)	(0.20)	(0.06)	(0.11)
Owns home	0.01	-0.03	0.01	-0.06	0.03	0.15	0.15	0.02	-0.13	0.12*	-0.14**
	(0.03)	(0.04)	(0.16)	(0.07)	(0.04)	(0.14)	(0.14)	(0.05)	(0.28)	(0.06)	(0.06)
Toilet	-0.06***	0.09***	0.04	-0.06**	0.04	-0.02	0.04	-0.06	-0.09	0.13**	0.02
	(0.02)	(0.02)	(0.07)	(0.02)	(0.03)	(0.04)	(0.08)	(0.04)	(0.17)	(0.05)	(0.04)
Livestock prod.	0.10***	-0.00	0.20	0.16***	0.17***	-0.05	0.15**	0.05	-1.00***	0.12***	0.03
	(0.02)	(0.02)	(0.13)	(0.02)	(0.03)	(0.07)	(0.07)	(0.04)	(0.20)	(0.05)	(0.04)
Off-farm income	-0.00*	-0.00	-0.00	-0.00	-0.00	-0.00	0.00	-0.00	-0.00**	-0.00*	0.00
	(0.00)	(0.00)	(0.00)	(0.00)	(0.00)	(0.00)	(0.00)	(0.00)	(0.00)	(0.00)	(0.00)
Country fixed effects	✓										
R^2	0.55	0.19	0.32	0.33	0.20	0.37	0.29	0.50	0.38	0.40	0.26
N	39219	7034	659	13040	7996	2042	1613	2054	884	1732	2165

Notes: Standard errors in parentheses
$*p < 0.10$, $**p < 0.05$, $***p < 0.01$.

Stochastic Production Frontier

Table S10 shows the results of the SPF estimations for the pooled sample of countries, first with a basic model that only includes the factors of production: the log of labor expenditure per ha, the number of family workers, and the log of farm size (column (1))[2]; in column (2) we add the log of field size as explanatory variable of the inefficiency term; in column (3) we additionally include demographic variables to the inefficiency term, column (4) adds terciles of land concentration and of the share of nonfarm labor at the regional level. Land concentration is calculated with the Herfindahl Hirschman Index (HHI), which measures the concentration of field size at the regional level. The share of nonfarm labor is calculated by adding the number of households whose primary activity is outside of agriculture (measured by less than 30% of total income coming from agriculture) relative to the total population at the regional level. Both variables are then divided in low, medium and high terciles. It should be noted that since i is the technical inefficiency term, a negative sign means a positive effect on technical efficiency.

In all specifications, the factors of production have the expected signs in most cases. Both variables for agricultural labor (labor costs per ha and family labor) increase land productivity. The stochastic frontier estimations also show

[2]We do not include proxies for input use or capital for the pooled sample of countries, as including these variables would decrease the sample size significantly. We do include these variables though for the country estimations when available (Table S9).

the inverse-relationship, as larger field sizes translate into lower land productivity. An increase of one percent in farm size decreases agricultural productivity per ha by 52%. Although the inverse-relationship holds, column (2), (3) and (4) show that farm size has a negative sign on the inefficiency function, meaning that an increase of farm size in fact increases technical efficiency. Once accounting for all factors of production and controlling the technical inefficiency function by demographic characteristics that may affect farm management, a greater farm size is correlated with a greater technical efficiency, or in other words, larger farms are closer to the stochastic production frontier than smaller farms.

In column (5) we add terciles for land concentration and the share of nonfarm labor at the regional level. The results show that a medium level of land concentration increases technical efficiency, but a high level of land concentration decreases technical efficiency, both relative to low levels of land concentration. A greater share of nonfarm labor at the regional level decreases technical efficiency, as expected because of the lower agricultural activity.

Table S10: Results of the Stochastic Production Frontier estimation for the pooled sample (Output variable: Log of land productivity)

	Basic model	Basic+ field size inefficiency function	Full specification	Full+ land concentration and density of nonfarm labor
Frontier				
Ln(Labor expenditure 2017PPP/HA)	0.06***	0.06***	0.06***	0.06***
	(0.00)	(0.00)	(0.00)	(0.00)
Family workers/ha	0.00***	0.00***	-0.00**	0.00
	(0.00)	(0.00)	(0.00)	(0.00)
Ln(Field size HA)	-0.52***	-0.55***	-0.55***	-0.54***
	(0.00)	(0.00)	(0.00)	(0.00)
Mu				
Ln(Field size HA)		-0.62***	-0.16***	-0.07**
		(0.10)	(0.04)	(0.03)
Years of education of the HH head			0.09***	0.02***
			(0.01)	(0.01)
Age of the HH head			0.02***	0.00***
			(0.00)	(0.00)
Only female adults			3.17***	2.28***
			(0.25)	(0.13)
Only male adults			2.70***	1.92***
			(0.23)	(0.13)
HHI land concentration Medium				-0.48***
				(0.06)
HHI land concentration High				0.32***
				(0.08)
Share nonfarm labor Medium				2.01***
				(0.12)
Share nonfarm labor High				2.97***
				(0.17)
Country fixed effects	✓	✓	✓	✓
R^2				
N	120404	120404	120404	120404

Notes: Standard errors in parentheses
$^{*}p < 0.10$, $^{**}p < 0.05$, $^{***}p < 0.01$.

To take a closer look at whether this relationship also holds for each country, Tables S11-S13 show the same specification as column (3) above for the pooled sample, and we add proxies for input use and capital when available for each country in the sample. As proxies for input use we include the costs of seeds per ha and other input costs per ha (fertilizers and chemicals) in logs, and as proxies for capital, we include owning mechanized equipment, irrigation

use, and the number of tropical livestock. Factors of production have the expected sign for all countries: there is a positive relationship between hired and family labor and land productivity for most countries; and there is also a positive relationship between input costs and capital ownership with land productivity, significant for most countries. The inverse-relationship also holds, and it is significant for most countries considered.

Farm size has a negative and significant coefficient on the inefficiency function for Burkina Faso, Cote d'Ivoire, Ecuador, Ethiopia, Georgia, Ghana, Guatemala, India, Iraq, Kenya, Kyrgyzstan, Malawi, Mozambique, Nepal, Nigeria, Peru, Rwanda, Tanzania, Timor-Leste, and Uganda; and a positive and significant coefficient for Bangladesh, Bulgaria, and Nicaragua. For most countries considered, after accounting for all factors of production, an increase in farm size translates into an increase of the farms' technical efficiency.

Table S11: Results of the Stochastic Production Frontier estimation for each country, Part I

	All	Albania	Armenia	Bangladesh	Bolivia	Bulgaria	Burkinafaso	CotedIvoire	Ecuador	Ethiopia	Georgia
Frontier											
Ln(Labor expenditure 2017PPP/HA)	0.06***	-0.00	-0.04***	0.03***	0.11***	0.13***	-0.01	0.05***	0.01	0.03***	0.00
	(0.00)	(0.01)	(0.01)	(0.00)	(0.02)	(0.03)	(0.01)	(0.01)	(0.01)	(0.01)	(0.00)
Family workers/ha	-0.00**	-0.00		-0.00	0.02***	0.00	0.05***	0.01	-0.01***	0.01	0.00**
	(0.00)	(0.00)		(0.00)	(0.01)	(0.00)	(0.00)	(0.01)	(0.00)	(0.01)	(0.00)
Ln(Field size HA)	-0.55***	-0.72***	-0.64***	-0.09***	-0.75***	0.24*	-0.63***	-0.74***	-0.99***	-0.50***	-0.99***
	(0.00)	(0.05)	(0.01)	(0.03)	(0.09)	(0.14)	(0.03)	(0.02)	(0.01)	(0.04)	(0.01)
Ln(Other input costs 2017PPP/HA)		0.03***		0.07***	0.07***	0.10***	0.03***		-0.00	0.07***	0.00
		(0.01)		(0.01)	(0.02)	(0.01)	(0.00)		(0.01)	(0.01)	(0.00)
Owns mechanized equipment		0.10**	0.10**	0.10***		0.70***	0.17***		0.07***	0.12	0.00
		(0.04)	(0.05)	(0.02)		(0.14)	(0.02)		(0.02)	(0.09)	(0.01)
Irrigation use		0.17***	0.23***							0.14***	
		(0.04)	(0.02)							(0.05)	
Tropical livestock units (TLU)		-0.01	0.00**	-0.00	-0.01			-0.00	-0.01***	-0.01	0.00*
		(0.01)	(0.00)	(0.01)	(0.01)			(0.00)	(0.00)	(0.01)	(0.00)
Ln(Seed costs 2017PPP/HA)				0.03***	-0.01	0.10***	-0.04***	-0.01	0.01	0.00	-0.00*
				(0.01)	(0.02)	(0.01)	(0.01)	(0.01)	(0.01)	(0.01)	(0.00)
Mu											
Ln(Field size HA)	-0.16***	-0.18	-4.14	0.65***	0.08	1.21***	-0.79***	-0.94***	-0.16***	-1.06***	-0.73***
	(0.04)	(0.50)	(5.89)	(0.19)	(0.15)	(0.14)	(0.04)	(0.08)	(0.03)	(0.27)	(0.06)
Years of education of the HH head	0.09***	-0.12		0.01	0.03	0.04***	0.02**	0.04***	0.03***		
	(0.01)	(0.14)		(0.01)	(0.02)	(0.01)	(0.01)	(0.01)	(0.01)		
Age of the HH head	0.02***	-0.02	-0.03	0.01	0.01	0.02***	0.00	-0.01*	0.00**	-0.01**	0.01**
	(0.00)	(0.03)	(0.06)	(0.00)	(0.01)	(0.00)	(0.00)	(0.00)	(0.00)	(0.01)	(0.00)
Only female adults	3.17***	-0.55	7.19	0.13	0.71***			0.96***	0.09	0.86***	0.33**
	(0.25)	(1.96)	(9.90)	(0.35)	(0.24)			(0.13)	(0.07)	(0.32)	(0.15)
Only male adults	2.70***	1.17	7.24	0.27	0.19			0.88***	-0.13	0.08	0.69***
	(0.23)	(2.30)	(10.07)	(0.70)	(0.33)			(0.13)	(0.10)	(0.50)	(0.21)
Country fixed effects	✓										
R^2											
N	120404	1295	1420	4010	387	796	7158	5657	7559	1770	4968

Notes: Standard errors in parentheses
$^{*}p < 0.10$, $^{**}p < 0.05$, $^{***}p < 0.01$.

Table S12: Results of the Stochastic Production Frontier estimation for each country, Part II

	Ghana	Guatemala	India	Iraq	Kenya	Kyrgyzstan	Malawi	Mali	Mozambique	Nepal	Nicaragua
Frontier											
Ln(Labor expenditure 2017PPP/HA)	0.00	0.04***	0.03***	0.03***	0.00	0.02***	0.04***	0.06***	0.09***	0.05***	0.09***
	(0.00)	(0.00)	(0.00)	(0.01)	(0.01)	(0.01)	(0.00)	(0.01)	(0.01)	(0.01)	(0.01)
Family workers/ha	0.02***	0.00**	0.00***	-0.01*	-0.01***	-0.02***	0.01***	0.02***	0.01**	0.02***	0.06***
	(0.01)	(0.00)	(0.00)	(0.00)	(0.00)	(0.01)	(0.00)	(0.01)	(0.00)	(0.01)	(0.02)
Ln(Field size HA)	-0.82***	-0.37***	-1.18***	-1.01***	-0.87***	-1.01***	-0.51***	-0.28***	-0.58***	-0.66***	0.23*
	(0.03)	(0.02)	(0.03)	(0.06)	(0.05)	(0.03)	(0.02)	(0.02)	(0.03)	(0.02)	(0.14)
Ln(Seed costs 2017PPP/HA)	0.02**	0.01	0.08***	0.04***	0.05***	0.01	0.02***	0.00		0.09***	
	(0.01)	(0.01)	(0.01)	(0.01)	(0.01)	(0.01)	(0.00)	(0.01)		(0.01)	
Ln(Other input costs 2017PPP/HA)	0.05***	0.06***	0.07***	0.13***	0.11***		0.07***	0.07***			
	(0.01)	(0.01)	(0.00)	(0.01)	(0.01)		(0.00)	(0.01)			
Owns mechanized equipment	0.11***	0.15***	0.13***			-0.15**	0.09***	0.32***		0.30***	0.07
	(0.02)	(0.02)	(0.01)			(0.06)	(0.03)	(0.04)		(0.04)	(0.12)
Irrigation use	-0.06		0.19***	-0.03	0.07	0.06	0.19***	0.73***	-0.01	0.10***	
	(0.09)		(0.01)	(0.06)	(0.04)	(0.04)	(0.02)	(0.07)	(0.04)	(0.03)	
Tropical livestock units (TLU)	0.05***	0.03	0.02**		0.06***	0.04***	0.03***	0.01***	0.05***	0.02***	
	(0.01)	(0.02)	(0.01)		(0.01)	(0.01)	(0.01)	(0.00)	(0.01)	(0.00)	
Mu											
Ln(Field size HA)	-0.96***	-0.23*	-1.10***	-0.65***	-0.82***	-2.19***	-0.49***	-0.07	-0.38***	-1.05***	1.14***
	(0.06)	(0.12)	(0.03)	(0.06)	(0.12)	(0.27)	(0.08)	(0.37)	(0.07)	(0.14)	(0.14)
Age of the HH head	-0.01***	-0.01*	-0.00***	-0.00**	-0.01***	0.02***	-0.01***	-0.13	0.01***	-0.01**	
	(0.00)	(0.01)	(0.00)	(0.00)	(0.00)	(0.01)	(0.00)	(0.14)	(0.00)	(0.00)	
Only female adults	0.40***	-0.82	0.05	0.05	0.56***	0.37	0.25***	11.94	0.52***	0.53***	0.53**
	(0.08)	(2.15)	(0.04)	(0.18)	(0.14)	(0.33)	(0.08)	(13.98)	(0.10)	(0.14)	(0.23)
Only male adults	0.87***	0.00	-0.00	0.40	0.34*	0.76	0.22	0.45	0.41***	1.49***	-0.01
	(0.10)	(0.20)	(0.08)	(0.34)	(0.19)	(0.81)	(0.14)	(15.96)	(0.15)	(0.33)	(0.29)
Years of education of the HH head		-0.05	-0.02***		-0.08***	0.03	-0.06***	1.33	-0.01	-0.02	
		(0.03)	(0.00)		(0.02)	(0.04)	(0.01)	(1.32)	(0.01)	(0.01)	
R^2											
N	6844	3515	13303	3278	6494	1749	8280	2072	6785	3688	880

Notes: Standard errors in parentheses
$*p < 0.10$, $**p < 0.05$, $***p < 0.01$.

Table S13: Results of the Stochastic Production Frontier estimation for each country, Part III

	Niger	Nigeria	Pakistan	Panama	Peru	Rwanda	Serbia	Tanzania	TimorLeste	Uganda	Vietnam
Frontier											
Ln(Labor expenditure 2017PPP/HA)	0.08***	0.04***	0.05***	0.28***	0.07***	0.09***	0.04***	0.08***	0.01***	0.01	0.05***
	(0.01)	(0.01)	(0.00)	(0.03)	(0.00)	(0.00)	(0.01)	(0.01)	(0.00)	(0.01)	(0.00)
Family workers/ha	-0.00	0.02***	0.02***	0.02	0.00**	-0.01***	-0.00	-0.02**	-0.00***	0.01	0.00***
	(0.01)	(0.01)	(0.00)	(0.01)	(0.00)	(0.00)	(0.02)	(0.01)	(0.00)	(0.01)	(0.00)
Ln(Field size HA)	-0.74***	-0.69***	-0.32***	-0.52***	-0.77***	-0.84***	-0.64**	-0.96***	-1.02***	-0.91***	-0.31***
	(0.04)	(0.04)	(0.02)	(0.18)	(0.02)	(0.02)	(0.28)	(0.06)	(0.00)	(0.08)	(0.02)
Ln(Seed costs 2017PPP/HA)	-0.01	-0.03***	0.02***	0.08	-0.00	-0.02***	-0.00	0.04***	0.05***	-0.02**	
	(0.02)	(0.01)	(0.01)	(0.06)	(0.00)	(0.00)	(0.02)	(0.01)	(0.00)	(0.01)	
Ln(Other input costs 2017PPP/HA)	0.10***	0.08***	0.11***	0.09***	0.06***	0.05***	0.04**	0.05***	-0.04***	0.11***	
	(0.02)	(0.01)	(0.01)	(0.03)	(0.00)	(0.00)	(0.02)	(0.01)	(0.00)	(0.02)	
Owns mechanized equipment	0.16**	0.13***				0.09	0.28***	0.25***	-0.01***	0.11**	
	(0.06)	(0.04)				(0.06)	(0.09)	(0.05)	(0.00)	(0.05)	
Irrigation use	0.17	0.05	0.31***			0.21***	0.17**	-0.02	-0.09***	0.05	
	(0.11)	(0.10)	(0.02)			(0.02)	(0.08)	(0.12)	(0.00)	(0.13)	
Tropical livestock units (TLU)	0.01***		-0.05***	-0.01	-0.00	0.15***	0.00	0.02***	-0.01***	-0.01	
	(0.00)		(0.01)	(0.01)	(0.00)	(0.01)	(0.01)	(0.01)	(0.00)	(0.01)	
Mu											
Ln(Field size HA)	-0.27	-0.88***	-1.48	0.07	-0.12**	-1.22***	-0.02	-0.52***	-0.34***	-0.71***	4.11
	(0.20)	(0.08)	(1.06)	(0.23)	(0.06)	(0.08)	(0.46)	(0.08)	(0.02)	(0.11)	(2.66)
Years of education of the HH head	0.07	0.01	0.10	0.00	0.04***		0.01	0.01	0.04***	-0.01	-0.70
	(0.06)	(0.01)	(0.08)	(0.02)	(0.01)		(0.02)	(0.01)	(0.01)	(0.01)	(0.49)
Age of the HH head	-0.00	0.01***	0.03	0.00	0.02***	-0.01***	0.01	0.00	0.01***	-0.01***	-0.01
	(0.01)	(0.00)	(0.03)	(0.00)	(0.00)	(0.00)	(0.01)	(0.00)	(0.00)	(0.00)	(0.03)
Only female adults	1.30	0.59***	0.75	0.20	1.20***	0.51***	0.54*	0.34***	0.27***	0.39***	6.32
	(0.92)	(0.16)	(0.85)	(0.36)	(0.18)	(0.08)	(0.31)	(0.07)	(0.10)	(0.09)	(4.51)
Only male adults	0.86	0.23	1.75	-0.29	0.45***	0.44***	0.08	0.22*	0.30***	0.32***	8.82
	(0.83)	(0.19)	(1.80)	(0.19)	(0.12)	(0.15)	(0.21)	(0.11)	(0.11)	(0.12)	(6.34)
R^2											
N	1649	2346	4399	970	8296	10504	666	1759	2217	2204	4802

Notes: Standard errors in parentheses
$*p < 0.10$, $**p < 0.05$, $***p < 0.01$.

References

W. M. Adams, R. Aveling, D. Brockington, B. Dickson, J. Elliott, J. Hutton, D. Roe, B. Vira, and W. Wolmer. Biodiversity conservation and the eradication of poverty. *science*, 306(5699):1146–1149, 2004.

C. Barrett, A. Travis, and P. Dasgupta. On biodiversity conservation and poverty traps. *Proceedings of the National Academy of Sciences*, 108(34):13907–13912, 2011.

J. B. Grace, T. M. Anderson, E. W. Seabloom, E. T. Borer, P. B. Adler, W. S. Harpole, Y. Hautier, H. Hillebrand, E. M. Lind, M. Pärtel, et al. Integrative modelling reveals mechanisms linking productivity and plant species richness. *Nature*, 529(7586):390–393, 2016.

T. Hasegawa, S. Fujimori, K. Takahashi, and T. Masui. Scenarios for the risk of hunger in the twenty-first century using shared socioeconomic pathways. *Environmental Research Letters*, 10(1):014010, 2015.

D. Leclère, M. Obersteiner, M. Barrett, and et al. Bending the curve of terrestrial biodiversity needs an integrated strategy. *Nature*, 585:551–556, 2020. doi: https://doi.org/10.1038/s41586-020-2705-y.

J. Liang, T. W. Crowther, N. Picard, S. Wiser, M. Zhou, G. Alberti, E.-D. Schulze, A. D. McGuire, F. Bozzato, H. Pretzsch, et al. Positive biodiversity-productivity relationship predominant in global forests. *Science*, 354(6309): aaf8957, 2016.

A. Popp, K. Calvin, S. Fujimori, P. Havlik, F. Humpenöder, E. Stehfest, B. L. Bodirsky, J. P. Dietrich, J. C. Doelmann, M. Gusti, T. Hasegawa, P. Kyle, M. Obersteiner, A. Tabeau, K. Takahashi, H. Valin, S. Waldhoff, I. Weindl, M. Wise, E. Kriegler, H. Lotze-Campen, O. Fricko, K. Riahi, and D. P. van Vuuren. Land-use futures in the shared socio-economic pathways. *Global Environmental Change*, 42:331–345, 2017. ISSN 0959-3780. doi: https://doi.org/10.1016/j.gloenvcha.2016.10.002.

M. Springmann, M. Clark, D. Mason-D'Croz, K. Wiebe, B. L. Bodirsky, L. Lassaletta, W. De Vries, S. J. Vermeulen, M. Herrero, K. M. Carlson, et al. Options for keeping the food system within environmental limits. *Nature*, 562 (7728):519–525, 2018.

D. Tilman, C. Balzer, J. Hill, and B. L. Befort. Global food demand and the sustainable intensification of agriculture. *Proceedings of the national academy of sciences*, 108(50):20260–20264, 2011.

M. van Dijk, M. Gramberger, D. Laborde, M. Mandryk, L. Shutes, E. Stehfest, H. Valin, and K. Faradsch. Stakeholder-designed scenarios for global food security assessments. *Global Food Security*, 24(100352), 2020. doi: https://doi.org/10.1016/j.gfs.2020.100352.

D. Williams, M. Clark, G. Buchanan, F. Ficetola, C. Rondinini, and T. David. Proactive conservation to prevent habitat losses to agricultural expansion. *Nature Sustainability*, 4:314–322, 2021.

S. Wirsenius, C. Azar, and G. Berndes. How much land is needed for global food production under scenarios of dietary changes and livestock productivity increases in 2030? *Agricultural systems*, 103(9):621–638, 2010.
